# A new specimen of *Plesiopterys wildi* reveals the diversification of cryptoclidian precursors and possible endemism within European Early Jurassic plesiosaur assemblages

**DOI:** 10.7717/peerj.18960

**Published:** 2025-03-31

**Authors:** Miguel Marx, Sven Sachs, Benjamin P. Kear, Mats E. Eriksson, Klaus Nilkens, Johan Lindgren

**Affiliations:** 1 Department of Geology, Lund University, Lund, Sweden; 2 Abteilung Geowissenschaften, Naturkunde-Museum Bielefeld, Bielefeld, Germany; 3 The Museum of Evolution, Uppsala University, Uppsala, Sweden; 4 Urwelt-Museum Hauff, Holzmaden, Germany

**Keywords:** Biodiversity, Diversification, Endemism, Jurassic, Plesiosaur

## Abstract

**Background:**

A virtually complete and articulated plesiosaur skeleton (MH 7) is described from the Lower Jurassic (Toarcian) Posidonienschiefer Formation near Holzmaden in southern Germany. Plesiosaur remains are rare in this rock unit compared to those of other marine reptiles, such as ichthyosaurs and thalattosuchian crocodylomorphs. The new specimen offers an opportunity to assess the biodiversity of Early Jurassic plesiosaurs documented from what is now Central Europe.

**Methods:**

The osteology of MH 7 is described and compared with other Early Jurassic plesiosaurs based on first-hand observations. Phylogenetic analyses using both equal weighting and weighted parsimony determined phylogenetic placement within Plesiosauria.

**Results:**

*Plesiopterys wildi* is an early-diverging plesiosauroid and a sister taxon to *Franconiasaurus brevispinus* and Cryptoclidia. MH 7 represents a subadult individual, providing an updated character state diagnosis of *Plesiopterys wildi*, which has hitherto only been known from the osteologically immature holotype SMNS 16812. The presence of multiple regionally distinct plesiosaur genera and species within the European epicontinental marine basins suggests possible paleobiogeographical segregation during the Toarcian.

## Introduction

From a global perspective, the Early Jurassic was characterized by the steady break-up of Pangea and associated climatic fluctuations that produced alternating greenhouse and icehouse conditions (Ruebsam et al., 2020; Nordt, Breecker & White, 2022). These paleoenvironmental changes coincided with the radiation of various reptile groups, including Plesiosauria (Brusatte et al., 2010; Benson, Evans & Druckenmiller, 2012; Toljagić & Butler, 2013; Puértolas-Pascual et al., 2021). The Lower Jurassic fossil record of plesiosaurians is especially diverse, with members of Plesiosauroidea Gray, 1825, Pliosauridae Seeley, 1874 and Rhomaleosauridae von Nopcsa, 1928 represented by numerous taxa from Europe, particularly in Germany and England (Benson, Evans & Druckenmiller, 2012; Benson & Druckenmiller, 2014).

The early Toarcian Posidonienschiefer Formation (Posidonia Shale) in southwestern Germany has been crucial for understanding the early evolution of Plesiosauria (Groβmann, 2007; Benson, Evans & Druckenmiller, 2012). A total of thirteen largely complete and articulated skeletons have been described to date (Sachs et al., 2025). These constitute five distinct genera and species: the plesiosauroids *Seeleyosaurus guilelmiimperatoris* (Dames, 1895), *Microcleidus brachypterygius* (von Huene, 1923), *Plesiopterys wildi* O’Keefe, 2004; the pliosaurid *Hauffiosaurus zanoni* O’Keefe, 2001; and the rhomaleosaurid *Meyerasaurus victor* (Fraas, 1910). However, the taxonomic status of some of these taxa remains contentious.

Groβmann (2007) assigned plesiosauroid plesiosaurs from the Posidonienschiefer Formation to two genera and species: *S*. *guilelmiimperatoris* and *M*. *brachypterygius*. O’Keefe (2004) alternatively established *P*. *wildi* based on an osteologically immature individual (SMNS 16812) that was subsequently assigned to *S*. *guilelmiimperatoris* by Groβmann (2007). Benson & Druckenmiller (2014) recovered *P*. *wildi* as the sister taxon of Cryptoclidia, and more derived than Microcleididae, thus rejecting this proposed synonymy. Vincent (2010) and Vincent et al. (2017a) also described osteologically immature individuals (SMNS 51143 and SMNS 51945) that they report as likely representing new taxa. Although, SMNS 51141 was referred to *M*. *brachypterygius* by Groβmann (2007).

Resolving the taxonomic status of the Posidonienschiefer Formation plesiosauroids is vital for reconstructing marine vertebrate biodiversity and paleobiogeography within the European Epicontinental Sea (which yields the most complete Lower Jurassic plesiosaur record globally; Tutin & Butler, 2017). In particular, the Toarcian plesiosaurs in Germany are distinguishable from coeval assemblages occuring in the Yorkshire Basin of England (Groβmann, 2007). The English Toarcian taxa include the rhomaleosaurids *Rhomaleosaurus cramptoni* (Carte & Baily, 1863), *Rhomaleosaurus thorntoni* Andrews, 1922, *Rhomaleosaurus zetlandicus* (Phillips in Annual report of the Yorkshire Philosophical Society, 1854), and *Sthenarosaurus dawkinsi* Watson, 1909; the microcleidids *Microcleidus homalospondylus* (Owen, 1865), and *Microcleidus macropterus* (Seeley, 1865), and the pliosaurids *Hauffiosaurus tomistomimus* Benson et al., 2011a, and *Hauffiosaurus longirostis* (Tate & Blake, 1876) (= *Macroplata* White, 1940: Benson et al., 2011a). These populations were paleogeographically separated in part by the extensive London-Brabant Massif, which may have instigated faunal endemism (Godefroit, 1994; Maisch, 1999; Maisch & Ansorge, 2004).

However, the marine reptile assemblages between these two regions should feasibly be similar, as is evident in their coeval actinopterygian fossil record (Wretman, Blom & Kear, 2016). Likewise, the Toarcian ichthyosaur fossil record across the European epicontinental basins is broadly similar with conspecific taxa present across different “zones” of the European Epicontinental Sea (Fischer, Guiomar & Godefroit, 2011).

*Microcleidus* has further been reported in other parts of the Central European Basin (CEB), including the area that is today Luxembourg (*Microcleidus melusinae* Vincent et al., 2017b) and the more southern Aquitarian Basin (*Microcleidus tournemirensis* (Bardet, Godefroit & Sciau, 1999)). These species variations can potentially be explained by temporal differences rather than paleobiogeographic segregation (Benson et al., 2011a), since *M*. *tournemirensis* derives from the late Toarcian and is therefore younger than *M*. *melusinae* (Vincent et al., 2017b). In addition, plausible remains of *S. guilelmiimperatoris* (GPIH unregistered) and *M*. *victor* (GPIH 4851), two plesiosaurs previously known only from the Southwestern German Basin, were reported also in Fennoscandian deposits (Sachs et al., 2016), suggesting a possible dispersal of these taxa northwards toward what is today Scandinavia. Sachs et al. (2025) determined that GPIH (unregistered) is instead only referrable to the family rank, Microcleididae. Finally, Toarcian ichthyosaur and plesiosaur genera long thought to be endemic to the CEB have been found in Siberia, indicating further range extensions into the polar regions (Zverkov, Grigoriev & Danilov, 2021). It is therefore surprising that plesiosaur taxa between the Yorkshire and Germanic basins are so disparate. In this article, we describe a new plesiosaur skeleton from the Posidonienschiefer Formation and establish its implications for plesiosaur assemblage segregation *vs*. synonymy within the Early Jurassic basins of Europe.

### Geological Setting

The Posidonienschiefer Formation has been extensively studied for over a century (see Röhl et al., 2001; Röhl & Schmid-Röhl, 2005; and Muscente et al., 2023 for an overview of the stratigraphy). The sediments of the Posidonienschiefer Konservat-Lagerstätte near Holzmaden, Germany, accumulated within the Southwestern German Basin as part of the shallow-marine epicontinental shelf that bordered the Tethys Ocean in the southeast during the Early Jurassic (Röhl et al., 2001; Röhl & Schmid-Röhl, 2005; Sinha et al., 2021). The marine environment was inundated with terrestrial clays and carbonates, leading to the formation of marls and marly shales (Röhl et al., 2001). The Posidonienschiefer Formation is divided into three ammonite zones, with the most complete plesiosaur remains being found at the top of the *Dactylioceras tenuicostatum* zone (εII_1_), and in both the lower and upper parts of the *Harpoceras serpentinum* zone (εII_3,_ εII_4_ & εII_9_) (Groβmann, 2006, 2007; Smith & Vincent, 2010; Vincent, 2010; Vincent et al., 2017a); (*H*. *serpentinum* zone = *Harpocerus falciferum* zone: Williams, Benton & Ross, 2015).

## Materials and Methods

MH 7 was originally excavated near the village of Holzmaden from ɛII_6C_ (*Harpoceras serpentinum* zone) in 1940. In 1970, MH 7 was considered for exhibition at Urwelt-Museum Hauff, but another plesiosaur (MH 8) was chosen instead and therefore took its place. MH 7 would remain in storage until it was decided by Rolf Hauff and Klaus Nilkens during the Covid-19 pandemic to prepare the skeleton. MH 7 was re-assembled from multiple shale slabs using historical notes on handwritten index cards as references. The fossil was prepared solely by Klaus Nilkens from Urwelt-Museum Hauff, mainly from the ventral side of the body, which originally faced downwards into the seafloor. Saws were used to cut the larger slabs into smaller portions and to reduce the thickness of the matrix. The cyanoacrylate, Kö-Kleber C2 (Kömmerling Chemische Fabrik GmbH, Pirmasens, Germany), was used in addition to epoxy during preparation. To remove the encasing rock matrix, hammers, chisels and small hand-held tools were used in addition to pneumatic air scribes and sandblasting with iron powder. The torso of MH 7 was enclosed in a hard carbonate concretion that needed to be approached with pneumatic chipping hammers and pneumatic air scribes. Incomplete and fractured bones were reconstructed with synthetic resin, epoxy clay, or with shale matrix.

Comparison of MH 7 with other Jurassic plesiosaurians is based on collection visits by M.M., B.P.K., S.S., and J.L. to Urwelt-Museum Hauff, Naturkundemuseum Stuttgart, Institut für Geowissenschaften der Universität Tübingen, Tübingen, Germany, and Museum für Naturkunde in Berlin, Germany. Phylogenetic analyses were performed using the matrix of Sachs, Eggmaier & Madzia (2024) (270 characters, 130 taxa) with parsimony analyses of equal and unequal weights and the addition of MH 7. Characters 25, 138, 139, 153, and 248 were previously modified by Madzia & Cau (2020) and thus differ from those provided by Benson & Druckenmiller (2014). For the Sachs, Eggmaier & Madzia (2024) matrix, 67 character states are ordered as per Madzia, Sachs & Lindgren (2019). Following Sachs, Eggmaier & Madzia (2024), *Neusticosaurus pusillus* (Fraas, 1881) was designated as the outgroup. TNT version 1.6 (Goloboff & Morales, 2023) was used to conduct the analyses (see the phylogenetic analysis section for specific search parameters). The STATS.RUN script calculated the consistency index, retention index, and recombined consistency index. This script comes with downloading TNT version 1.6. Bremer indices were calculated directly from the TNT v.1.6 interface (Trees/Bremer Supports). For the weighted analyses, symmetric resampling was used to assess node support using the same parameters as Sachs, Eggmaier & Madzia (2024) with symmetric resampling using the settings: traditional search, 1,000 replicates, default change probability (33), default settings for the cutoff (collapse groups below 1) and frequency differences (GC) as output. Eight character changes were made to the holotype of *Plesiopterys wildi* (SMNS 16812) based on first-hand study of the specimen (characters 55, 83, 151, 153, 192, 215, 257, and 260). See the supplemental for updates to these character changes.

## Results

SYSTEMATIC PALEONTOLOGY

Sauropterygia Owen, 1860

Plesiosauria de Blainville, 1835

Plesiosauroidea Gray, 1825

*Plesiopterys* O’Keefe, 2004

*Plesiopterys wildi* O’Keefe, 2004

**Holotype.** SMNS 16812; an osteologically immature skeleton (O’Keefe, 2004). Many of the vertebral neural spines, centra and ribs have been reconstructed, as have sections of the pectoral and pelvic girdles, and phalanges.

**Figure 1:**
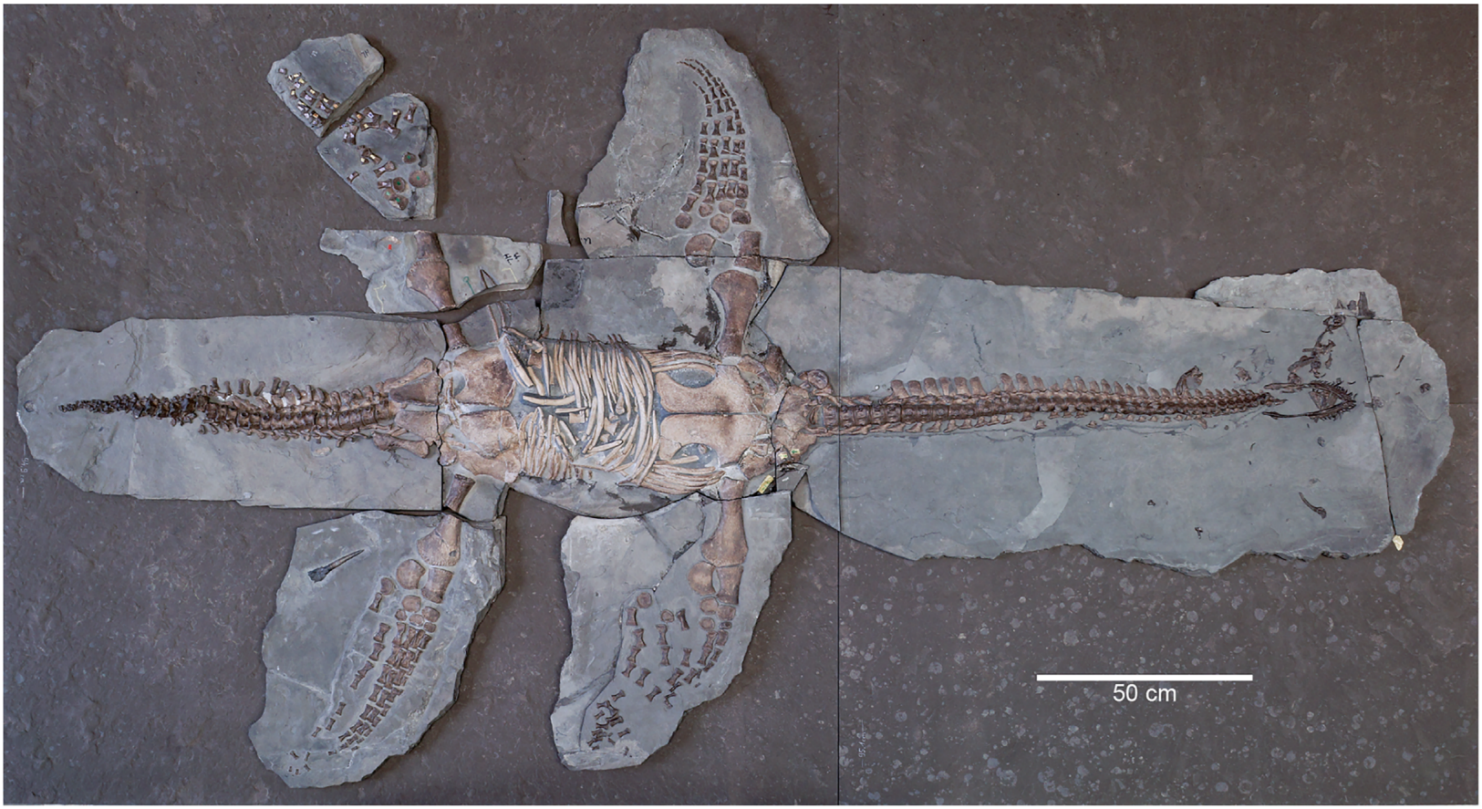
Skeleton of MH 7 exposed in ventral view.

**Referred specimen.** MH 7, A ~3-m long plesiosauroid skeleton (Fig. 1) lacking only the distal phalanges along with some braincase elements and parts of the craniofacial skeleton, which are either missing or still embedded in matrix. The following cranial elements are missing: anterior ramus of the right squamosal, one or both postorbitals, the postfrontals, prefrontals, one of the jugals, the anterior portion of the right pterygoid, left pterygoid, vomers may or may not be present, one of the ectopterygoids, one or both of the palatines, the left hyoid, the right prootic and right exoccipital-opisthotic.

**Type locality and horizon.** εII_4_ in the lower Toarcian Unterer Schiefer (*Harpoceras serpentinum* zone) from the Posidonia Shale Formation near Holzmaden, Germany, as reported by O’Keefe (2004).

**Locality and horizon of referred specimen.** MH 7 was excavated from εII_6C_ of the lower Toarcian Posidonienschiefer Formation. The provenance quarry was located in a forest near Holzmaden, between the villages of Schlierbach and Ohmden.

**Revised diagnosis.** A plesiosauroid plesiosaurian with the following autapomorphies: An elongated groove for the internal carotid artery on the dorsal surface of the quadrate ramus of the pterygoid (present in MH 7); a short medial flange of the pterygoid separating the posterior interpterygoid vacuity from the anterior interpterygoid vacuity (present in MH 7 and SMNS 16812); star-shaped interclavicle formed by a deeply concave anterior margin, two anterolateral processes, a second pair of processes oriented laterally, and one posteriorly oriented process (present in MH 7 and SMNS 16812). The following combination of characters further differentiates the taxon from all other plesiosaurians: exposure of the cultriform process of the parasphenoid to the posterior margin of the anterior interpterygoid vacuity (present in SMNS 16812 and inferred in MH 7), possession of a narrow and straight quadrate ramus of the pterygoid (present in both specimens); tall dorsal ramus of the maxilla, demarcating the anterior margin of the orbit (present in both specimens); lack of supernumerary elements in the limb (present in both specimens); lack of a proximal flange on the radius (present in both specimens); posterior-half of caudal neural spines are inflected posteriorly (present in both specimens).

**Description**

**Skull**

**Figure 2:**
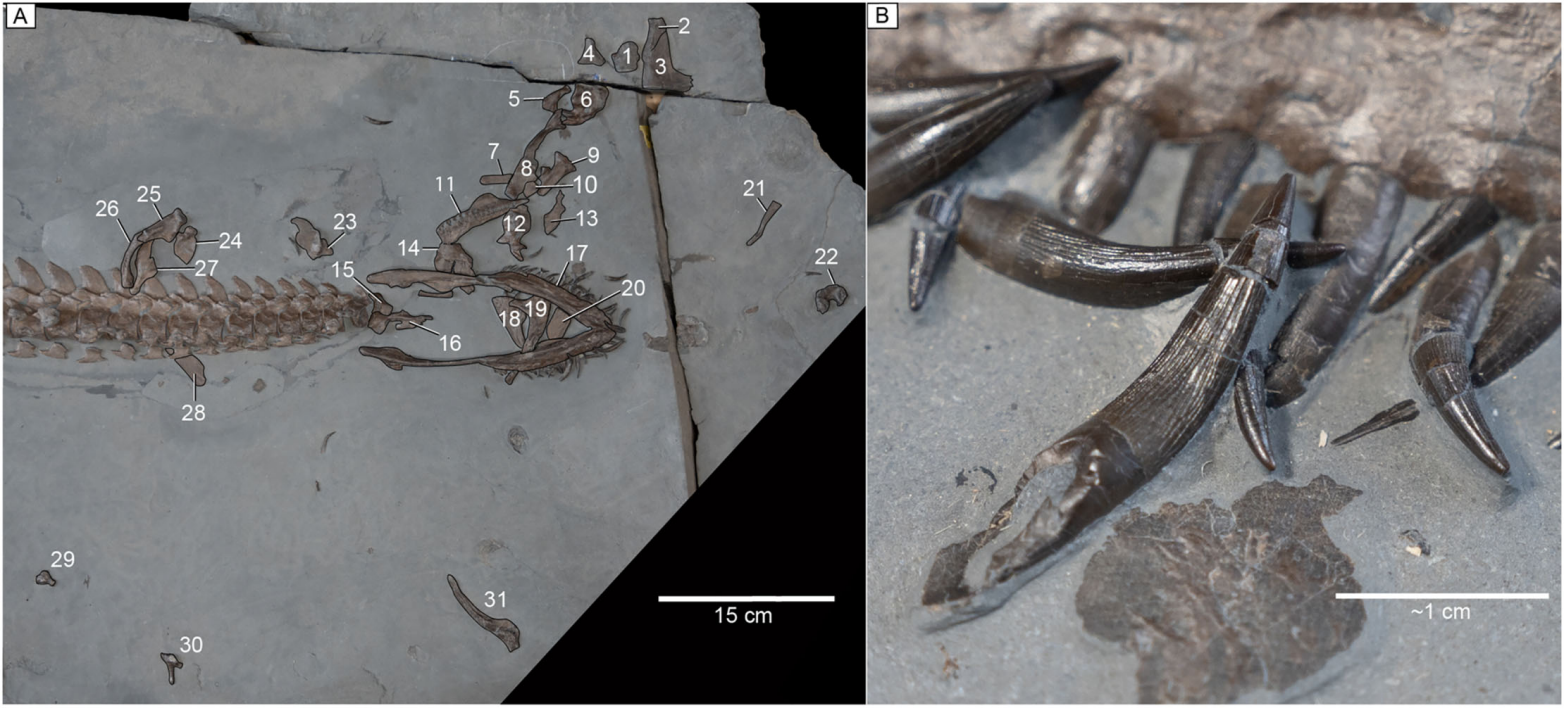
Skull bones and dentition of MH 7. (A) Map of skull bone elements. Bone map key: 1, basioccipital; 2, left quadrate; 3, partial left squamosal; 4, left postorbital?; 5, unidentified element; 6, atlas-axis complex; 7, unidentified element; 8, left temporal bar (partial left squamosal); 9, parabasisphenoid; 10, unidentified element; 11, left maxilla; 12, anterior cervical vertebra; 13, frontals; 14, partial left maxilla and perhaps part of the palate; 15, unidentified element; 16, vomers?; 17, mandible; 18, right premaxilla; 19, right maxilla; 20, possible palatine or jugal; 21, unidentified element; 22, supraoccipital; 23, ectopterygoid; 24, right quadrate flange of the pterygoid; 25, right quadrate; 26, right squamosal suspensorium; 27, parietals; 28, jugal; 29, left prootic; 30, left exoccipital-opisthotic; 31, right partial pterygoid. (B) Close-up image of dentition showing apicobasally extending ridges along the lingual face of the teeth but absent on the labial.

**Figure 3:**
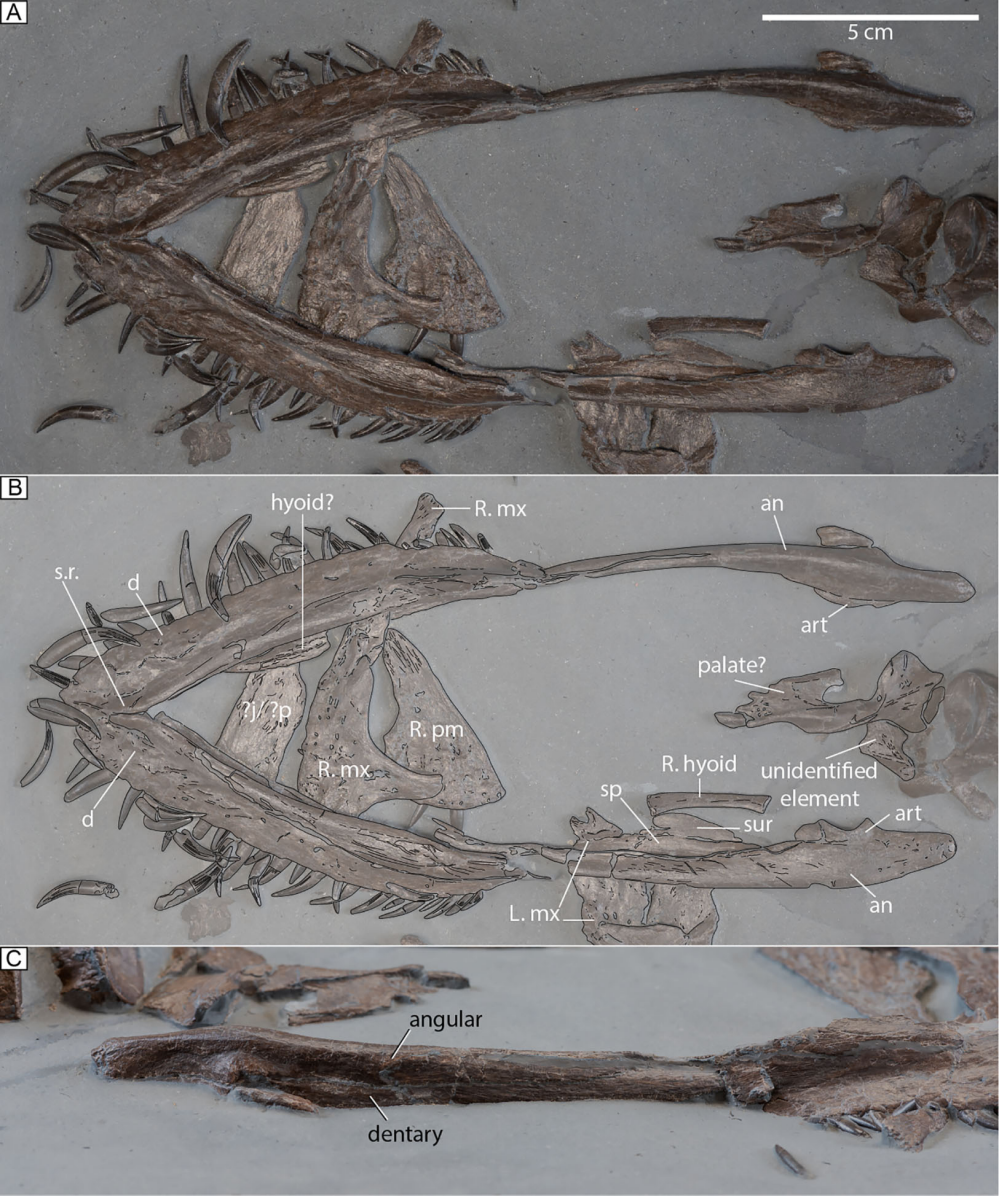
Mandible and skull elements of MH 7. (A) Ventral view of the mandible and associated skull elements. (B) Mandible and skull elements labeled. (C) Left lateral view of the mandible. Abbreviations: an, angular; art, articular; d, dentary; j, jugal; R. mx, right maxilla; L. mx, left maxilla; p, palatine; R. pm, right premaxilla; sp, splenial; s.r., symphyseal ridge; sur, surangular.

**Craniofacial skeleton and palate.** Much of the craniofacial skeleton is disarticulated and dispersed throughout the matrix at the anterior end, while the mandible is intact (Fig. 2A). Isolated teeth are also dispersed throughout the matrix, but many remain in their original position in the mandible (Fig. 2B). The right premaxilla has been distorted by dorsoventral flattening; thus, its original shape cannot be accurately determined. However, as preserved, the premaxilla is broad anteriorly and then narrows posteriorly. Medially, this element exhibits an elongated and straight contact with its counterpart (Figs. 3A, 3B). The external surface is marked by indentations on the bone.

**Figure 4:**
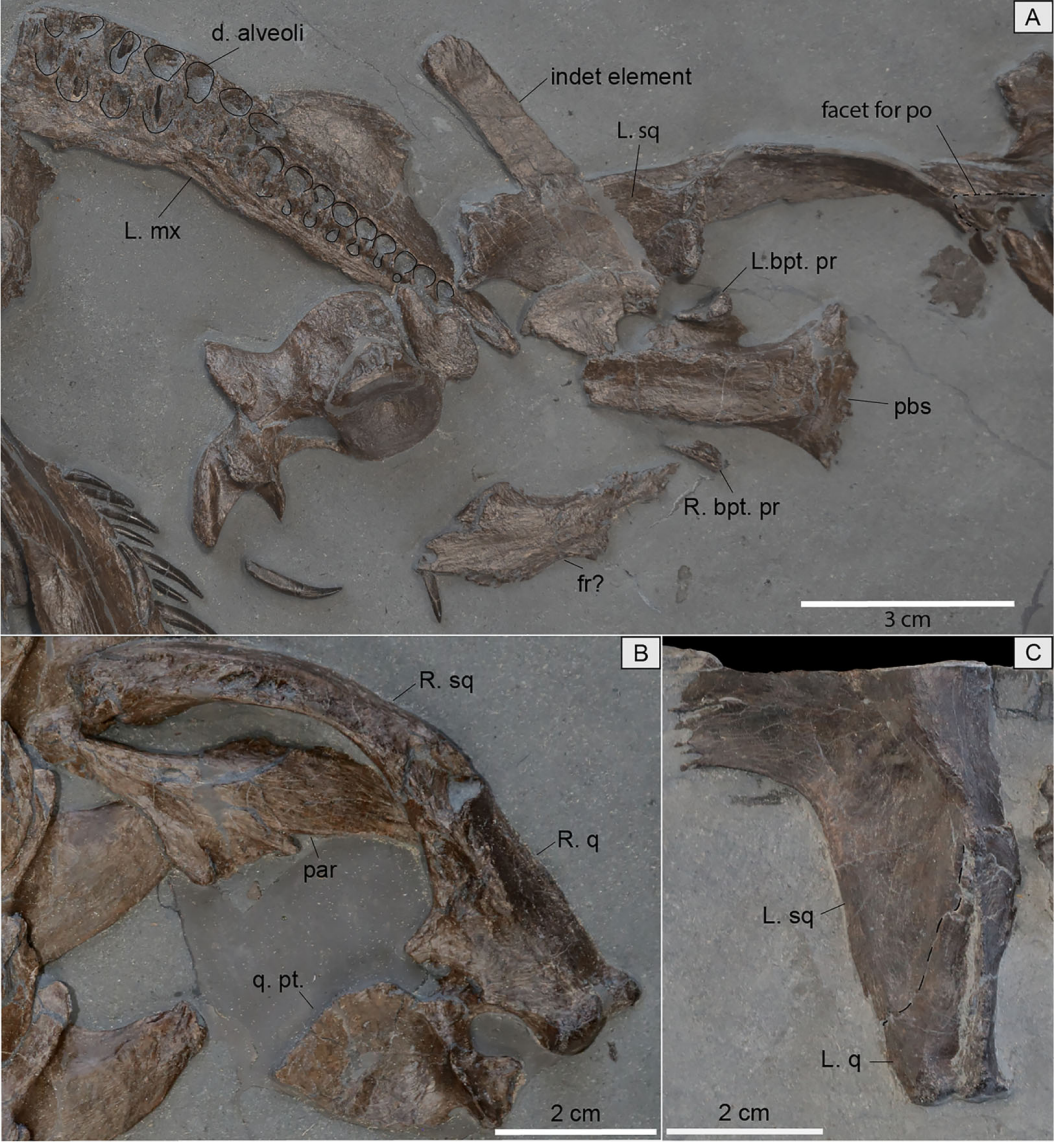
Isolated skull elements of MH 7. (A) Close-up image with exposed dental alveoli and replacement alveoli of the left maxilla. Plausible frontals are also in view along with the parabasisphenoid in ventral view, part of the left squamosal, and an unidentified element. Also in view is the left temporal bar with a facet for the postorbital located anteriorly. (B) Right suspensorium, right quadrate, right quadrate flange of the pterygoid, and parietals. (C) Part of the left squamosal in lateral view along with an articulated left quadrate. Abbreviations: R./L. bpt. pr, Right/Left basipterygoid process; d, dental; fr, frontal; mx, maxilla; par, parietal; pbs, parabasisphenoid; po, postorbital; q, quadrate; q. pt., quadrate process of the pterygoid; sq, squamosal.

A dorsal ramus from the maxilla demarcates the anterior margin of the orbit (Figs. 3A, 3B). The bone surface of the maxilla is pitted by foramina. Posteriorly, the maxilla tapers to form a low bar. Sixteen dental alveoli are visible in the left maxilla (Fig. 4A). Medially to these, replacement dental lamina foramina are apparent. In lateral view, a sheet of bone is present on the left maxilla that rises dorsal to the upper tooth row.

A jugal is identified among the many dispersed cranial elements in the matrix, as it preserves part of the orbital rim (Fig. 2A). Part of the left temporal bar is preserved; it is mediolaterally compressed with a facet for the postorbital present anteriorly (Fig. 4A). A pair of plausible frontals is exposed and would have formed a smooth surface across the skull roof between the orbits (Fig. 4A). The dorsal ramus of the right squamosal is visible and compressed anteroposteriorly (Fig. 4B); its apex is low and flat. A medially placed groove extends along almost the entire length of the ramus. Part of the left squamosal is also visible where it articulates with the quadrate and partly contributes to the temporal bar (Fig. 4C). The entire contribution of the squamosal to the temporal bar cannot be assessed; nonetheless, the squamosal makes a small contribution to the ventral margin of the temporal bar with an interdigitating suture visible laterally (Fig. 4C).

The condyles of the quadrate are exposed distally. The medial condyle is well-rounded while the lateral one is flatter (Fig. 4B). Along the medial surface of the bone, a facet to receive the quadrate process of the pterygoid is evident (Fig. 4B).

**Figure 5:**
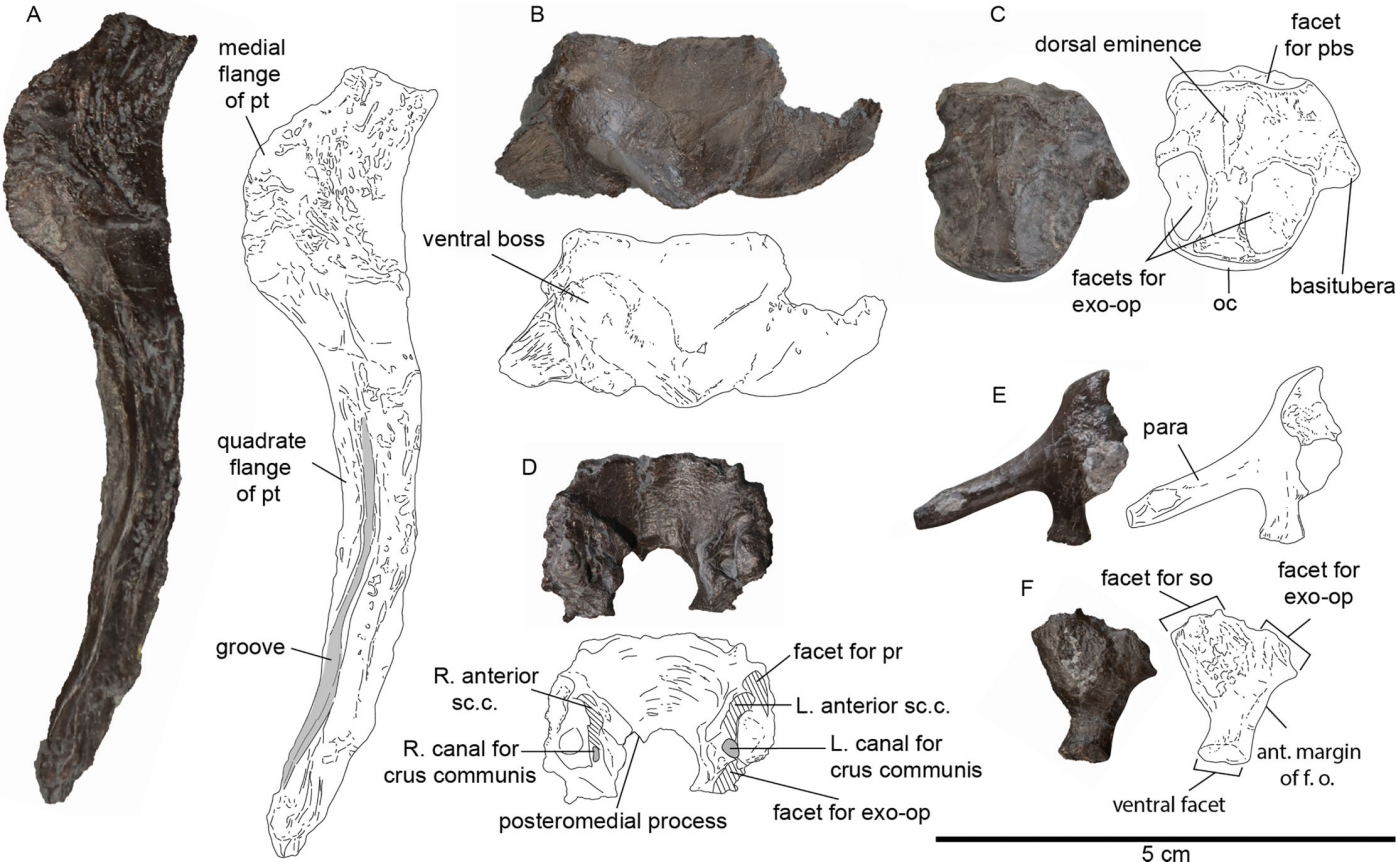
Palate and braincase elements of MH 7. (A) Dorsal view of partial right pterygoid. The gray region highlighted on the bone denotes the groove on the dorsal surface. (B) Ectopterygoid in view with a ventral boss. (C) Basioccipital in dorsal view. (D) Supraoccipital in ventral view. (E) Left exoccipital-opisthotic in posterior view. (F) Lateral view of left prootic. Abbreviations: exo-op, exoccipital-opisthotic; f.o., fenestra ovalis; oc, occipital condyle; pbs, parabasisphenoid; pr, prootic; pt, pterygoid; sc.c., semi-circular canal; so, supraoccipital.

From the few observable palatal bones, the posterior portion of the right pterygoid is preserved and exposed in dorsal view (Fig. 5A). An elongated groove for the internal carotid artery extends along the dorsal surface of the narrow and straight quadrate flange. The latter forms an extensive contact along its articulating surface with the quadrate (Fig. 4B). A short and rounded medial flange from the pterygoid is apparent where the posterior interpterygoid vacuity would have terminated anteriorly (Fig. 5A).

In MH 7, the anterior portion of the pterygoid narrows following this flange. Thus, the anterior-most portion of the parasphenoid would have been close to the posterior opening of the anterior interpterygoid vacuity, likely contributing to its posterior margin. Additional palatal elements include an ectopterygoid with a ventral boss (Fig. 5B), and a possible palatine (Figs. 2A, 3B).

**Braincase and hyoid.** A parabasisphenoid is visible in ventral view and has a smooth ventral surface that is concave (Fig. 4A). The parasphenoid covers most of the ventral surface without the presence of medial foramen exposing the basisphenoid. A foramen for the passage of the carotid artery could not be confidently identified and is likely obscured by matrix. The basipterygoid process forms a buttress with a smooth ventral surface and a broad lateral facet for the pterygoid (Fig. 4A). Posteriorly, the parabasisphenoid broadens significantly to receive the basioccipital.

The basioccipital exhibits a rounded occipital condyle outlined by a ridge that contacts the facets for the exoccipital-opisthotics (Fig. 5C). Matrix covers part of the occipital condyle, thus obscuring this surface. Facets for the exoccipital-opisthotics are separated and have ellipsoidal impressions on the dorsal surface that are slightly greater than half the length of the basioccipital (Fig. 5C). An eminence on the dorsal surface has a triangular outline and tapers posteriorly to terminate almost half-way along the paired exoccipital-opisthotic facets. Most of the anterior surface of the basioccipital is not visible; however, a sulcus located medially may delineate the notochordal pit.

The ventral surface of the supraoccipital is exposed (Fig. 5D). Ventromedially, the surface of this element is smooth. Posteromedially, a short and sharply tapering process is visible pointing posteriorly. The anterior semicircular canal, and crus communis are most visible in the left-half of this element (Fig. 5D). Facets for the prootic and exoccipital-opisthotic occur anteriorly and posteriorly, respectively.

The left exoccipital-opisthotic is preserved; however, only the posterior surface of this complex is visible (Fig. 5E). The paraoccipital process is slightly compressed dorsoventrally and extends ventrolaterally to a blunt end. The left prootic is also preserved and evinces a short contact with the basisphenoid, with a smoothly curved posterior margin that contributed to the fenestra ovalis. Dorsally, this element would have partly articulated with the supraoccipital and the exoccipital-opisthotic (Fig. 5F). The medial face of the prootic in MH 7 is obscured by sediment, thus the vestibule and foramina for cranial nerves cannot be described.

A well-preserved right hyoid is visible between the rami of the mandible, close to its life position (Fig. 3B). This element is slightly curved in its morphology.

**Mandible.** The ventral surface of the dentary has a rough texture with numerous foramina piercing the surface (Figs. 3A, 3B). The symphysis is elaborated by a keel that becomes shallow anteriorly (Fig. 3B). An exact count is not possible, however, the maximum number appears to be close to thirty, as ~26 teeth are visible within the right dentary or close to their original position in this element, and ~31 teeth are visible within and or near the left dentary. The angular contacts the dentary and articular. The mandibular rami are straight and not bowed laterally. The retroarticular processes of MH 7 are oriented slightly posteromedially with a gentle posterodorsal inclination.

The splenial is an elongated element that extends for most of the entire length of the mandible, but its contribution to the symphysis cannot be assessed. The surangular forms a tall apex that contributes significantly to the coronoid process. The coronoid is not observable and matrix obscures any fenestrae on the medial side of the mandible. In lateral view, the mandible has a smooth surface near the mandibular glenoid with a clear suture between the dentary and angular (Fig. 3C).

**Dentition.** The tooth crowns are all recurved. They are elongated and oval in cross-section with labio-lingual compression (Fig. 2A). The crowns are ornamented by narrow ridges extending apicobasally along the lingual face but also on the mesial and distal surfaces, whereas the labial face is smooth. The largest teeth occur mesially with subsequent size reduction distally.

**Axial skeleton**

**Figure 6:**
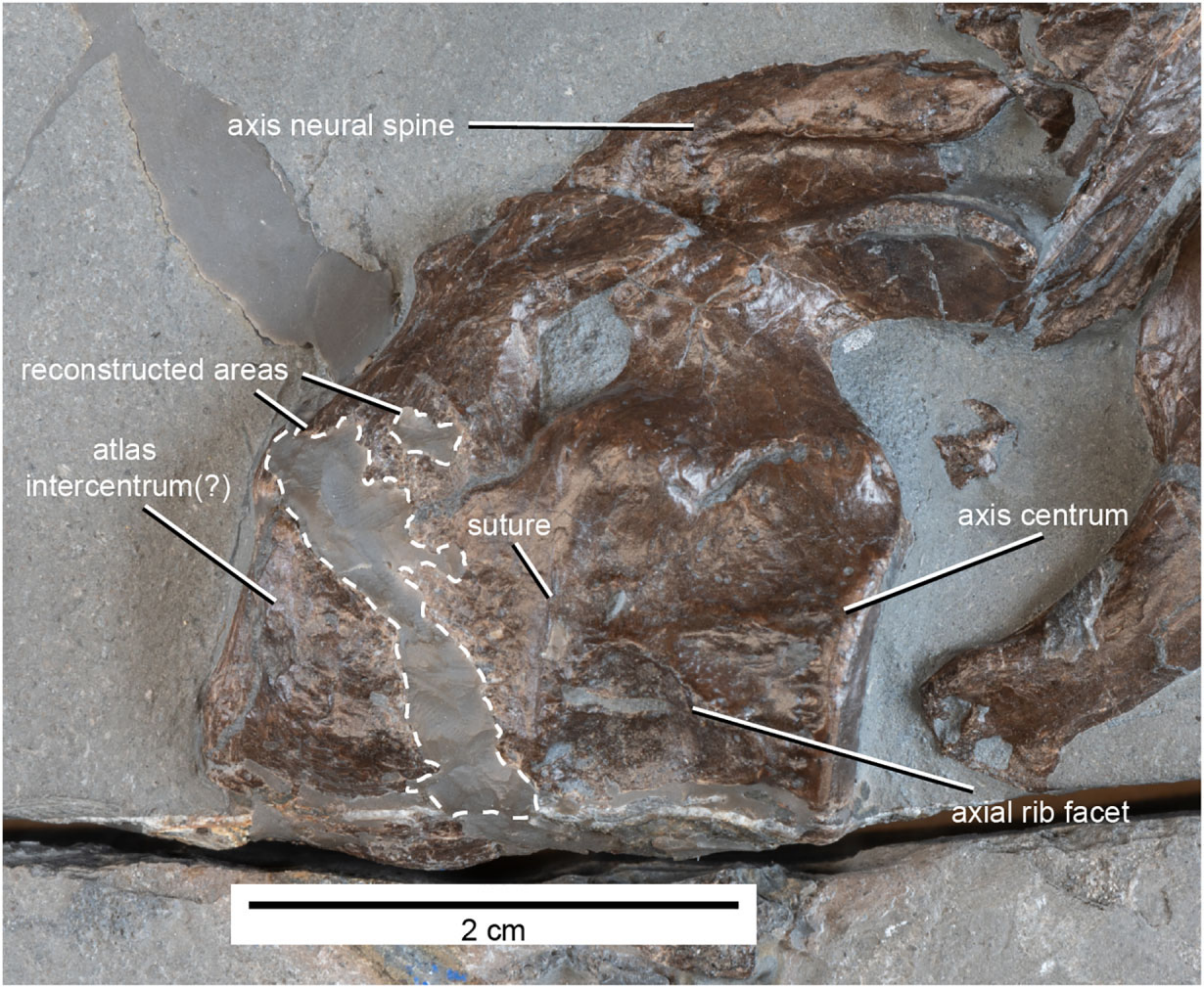
Atlas-axis complex of MH 7 in left lateral view.

**Cervical vertebrae.** Thirty-five cervical vertebrae remain in articulation. The atlas-axis complex and one additional cervical vertebra are dislocated from the anterior end of the cervical column, giving a total cervical vertebra count of 38. The axis centrum and what appears to be the atlas intercentrum are clearly distinguishable by a vertical suture running along the lateral surface (Fig. 6). The neural arch of the atlas is short dorsoventrally and contacts the axial neural arch posteriorly. The neural spine of the axis is low but elongated and becomes transversally wider posterodorsally along its apex. The axial neural spine becomes more compressed before merging ventrally with the postzygapophyses. The lateral surface of the axis is smoothly concave before meeting the facet for the axial rib. The posterior articular facet of the axis is platycoelous. Damage along the ventral surface of the atlas-axis complex prevents interpretation of a hypophyseal ridge.

**Figure 7:**
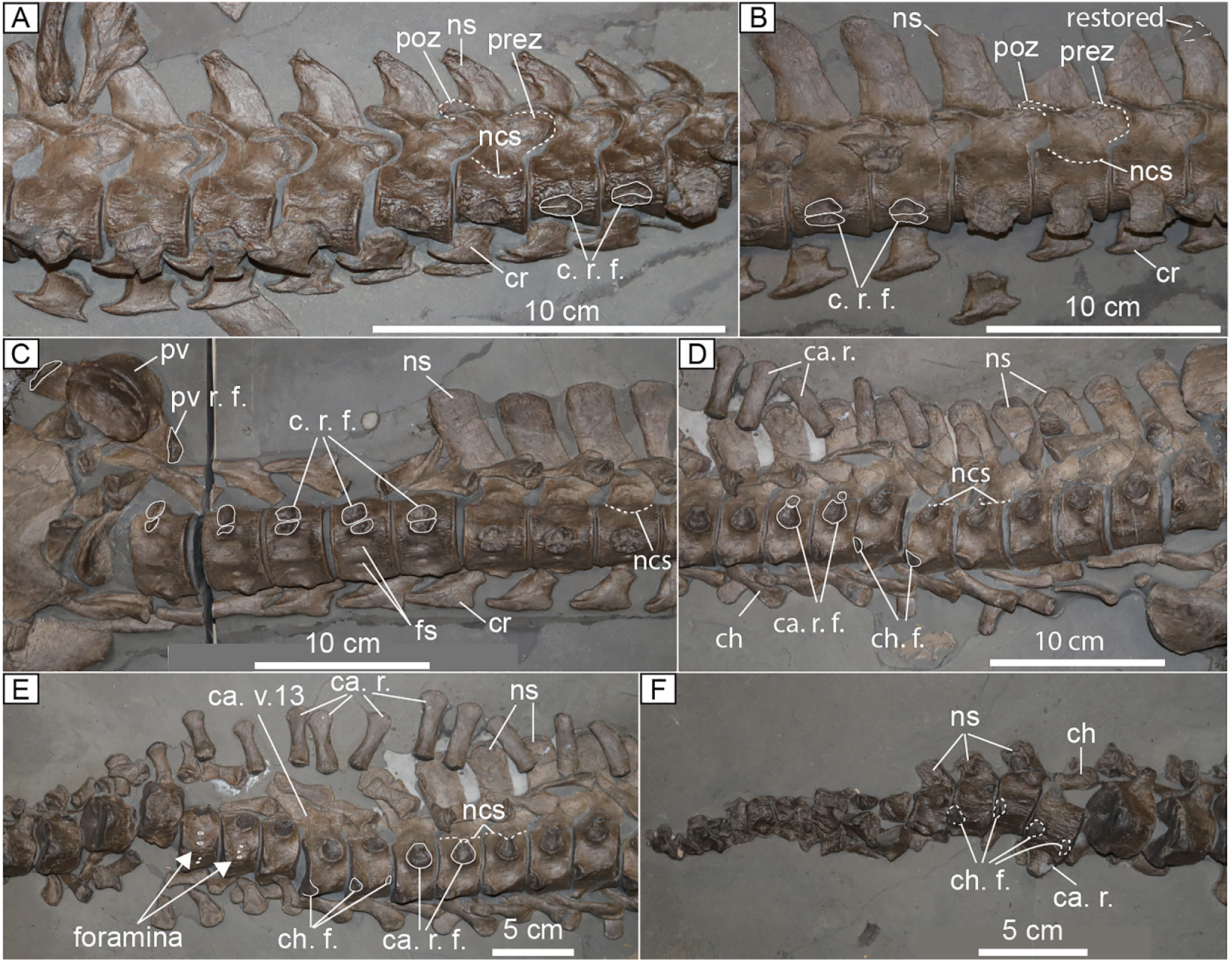
Axial skeleton of MH 7. (A) Anterior cervical vertebrae. (B) Middle cervical vertebrae; (C) Posterior cervical vertebrae and a single exposed pectoral vertebra. (D) Anterior caudal vertebrae. (E) Middle caudal vertebrae. (F) Posterior caudal vertebrae. Abbreviations: ca. r., caudal rib; ca. r. f., caudal rib facet; ca. v. 13, caudal vertebra 13; cr, cervical rib; c. r. f., cervical rib facet; ch, chevron; ch. f., chevron facet; fs, foramina subcentralia; ncs, neural central suture; ns, neural spine; prez, prezygapophyses; poz, postzygapophyses; pv, pectoral vertebra; pv r. f., pectoral vertebra rib facet.

Most of the cervical vertebrae are slightly longer than high (Table S1). The neural arches of the cervical vertebrae are completely fused to their adjoining centra with a U-shaped neurocentral suture visible in lateral view (Figs. 7A–7C). A rugose bone texture is observable on the lateral and ventral surfaces of the cervical centra, close to the articular rims (Fig. 7A). The articular facets of the centra are obscured in almost all of the cervicals, aside from the first two postaxial vertebrae, which exhibit amphicoelous facets. All the cervical ribs are unfused. The pre- and postzygapophyses are oriented horizontally along the entire cervical series. The rib facets are all double-faceted and co-joined in most of the cervical vertebrae. The interface between the facets is delineated by a narrow ridge in the anterior vertebrae that eventually inverts to form a groove in the posterior cervical vertebrae. The rib facets in MH 7 are ellipsoidal in shape for almost all of the cervical vertebrae, except for the posterior-most in which the diapophyses and parapophyses are more circular in profile (Fig. 7C). The diapophyses have a slightly greater surface area than the parapophyses in the cervical vertebrae. Rib facets for the last four cervical vertebrae in the series are oriented posterolaterally.

Nearly all the cervical vertebrae exhibit ribs that are hatchet-shaped with a more pronounced posterior process on the distal end (Figs. 7A, 7B). The posterodistal process of the cervical rib becomes increasingly more elongated moving posteriorly along the neck. The posterodistal process forms a cylinder in the posterior-most cervical vertebrae (Fig. 7C). A pair of foramina on the ventral surface of the cervical centra is separated by a subtle ventral ridge in the posterior cervical vertebrae (Fig. 7C). The middle and anterior cervical vertebrae lack the mid-ventral ridge separating the foramina. A lateral longitudinal ridge is absent in all the cervical vertebrae except for perhaps postaxial cervical vertebrae four and five where it is possible an incipient ridge is present; however, this possible ridge may be an artefact created by the rugose texture of bone on the lateral surface. Thus, it is not certain that this trait is present in MH 7.

The shape of the neural spine varies along the cervical series. The anterior cervical vertebrae have neural spines that are curved posteriorly and an anteroposteriorly elongated base that tapers distally, forming a shark fin-shaped neural spine. The neural spines become more rectangular in the posterior-half of the neck and are tall relative to the centra with decreased spacing between adjacent neural spines (Fig. 7C).

**Pectoral and dorsal vertebrae.** A pectoral vertebra displaced from the vertebral series is visible on the right side of the interclavicle in MH 7 (Fig. 7C). The rib facet is dorsoventrally expanded and oriented ventrolaterally. The articular facet of the centrum is platycoelous. The subsequent pectoral vertebra is mostly obscured by matrix. The dorsal vertebrae are completely obscured by matrix, aside from the ventral surface of four dorsal centra poking through the gastral basket. In what is exposed, these centra exhibit an hourglass shape in ventral view with platycoelous articular facets.

**Caudal vertebrae.** At least 35 to 36 caudal vertebrae are visible in near perfect articulation, mostly in right lateral view (Figs. 7D–7F). An exact count is difficult, as the last several vertebrae are slightly disarticulated and partially covered by matrix. The total number of caudal vertebrae is unlikely to have been much more than 36 because the anterior-most caudal in the series exhibits a dorsoventrally expansive rib facet, similar to a sacral vertebra. In lateral view, the neural arch contributes to the caudal rib facet (Fig. 7D). In ventral view, foramina subcentralia are apparent (Fig. 7E). The neural spines of the anterior 10 caudal vertebrae in the series exhibit tall neural spines that are approximately 1.5 times greater in height than the centra with rounded distal ends (Fig. 7D). The neural spines of the posterior-most caudal vertebrae significantly shorten (Figs. 7E, 7F) and the last caudal vertebrae are difficult to discern as their associated neural arches, ribs, and chevrons are disarticulated and intermingled with the centra.

**Figure 8:**
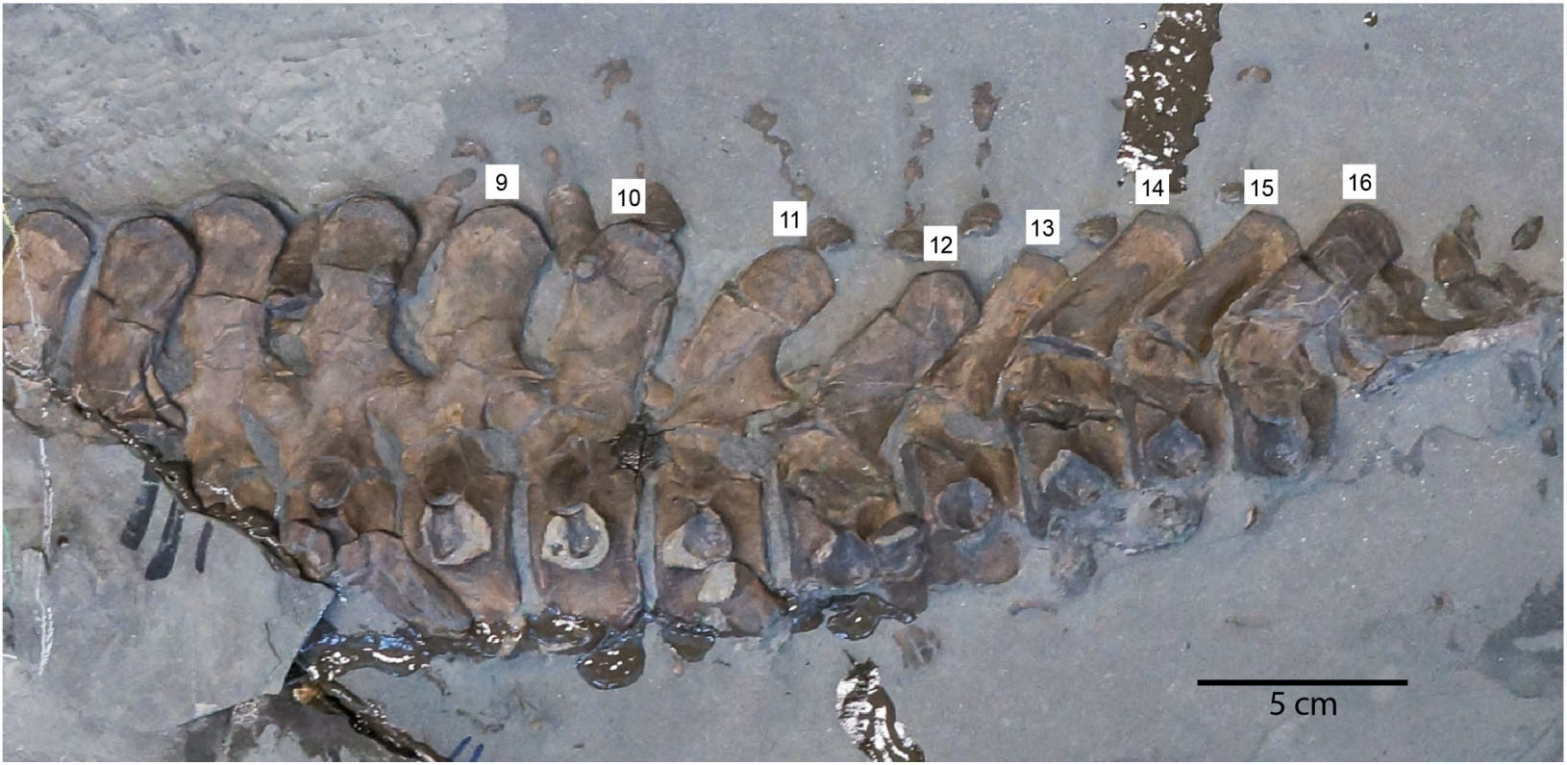
Caudal vertebrae of MH 7 in left lateral view; vertebrae positions are labeled above the neural spines. Note the posterior inflection of the neural spines as the series shifts to the posterior-half of the tail.

None of the caudal ribs and chevrons are fused to the centra. The first four caudal vertebrae in the series exhibit facets for the chevrons that do not protrude far from the centra relative to the sequential caudal vertebrae where these facets project further from the ventral surface of the centrum. The articular facets of caudal centra 18–21 are hexagonal in shape. The caudal ribs are dorsoventrally compressed and expand distally. The hemapophyses of MH 7 are elongated in the anterior portion of the caudal series and become shorter posteriorly with expanded proximal and distal ends. The 11th caudal vertebra in the series of MH 7 exhibits a more posteriorly inflected neural spine as opposed to the preceding vertebrae (Fig. 7E). From the 13th caudal vertebra to the 16th, the neural spines are inflected posteriorly at a low angle (Figs. 7E, 8).

**Figure 9:**
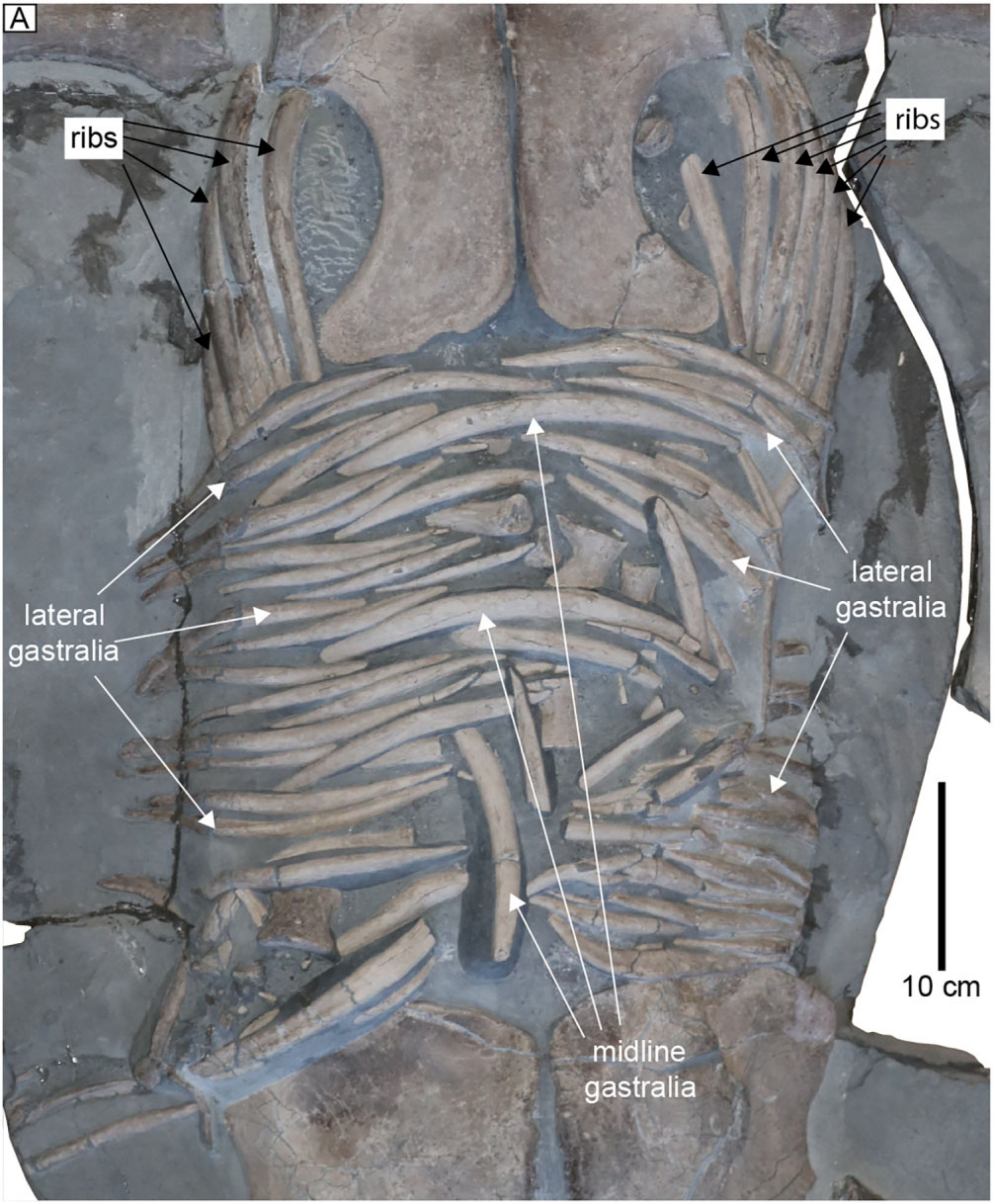
Gastral basket of MH 7; some dorsal ribs remain near life position in the anterior portion of the trunk. A minimum of three rows for the gastralia are apparent; two laterally and one medially.

**Ribs and gastralia.** The bodies of the anterior dorsal ribs are exposed in MH 7. These are all circular in cross-section and oriented posteriorly (Fig. 9); in life they would therefore have had at least a slight posterior inclination when articulated. The gastral basket is a tightly interlocking mesh of ~60 spindle-shaped gastralia arranged in at least three rows (Fig. 9). The gastralia with the greatest thickness lay along the midline. Smaller and narrower gastralia line the ventrolateral margins of the trunk (Fig. 9). Approximately 26 gastralia lie in the right row, while ~10 are aligned in the middle, and ~22 are present in the left row (Fig. 9).

**Appendicular skeleton**

**Figure 10:**
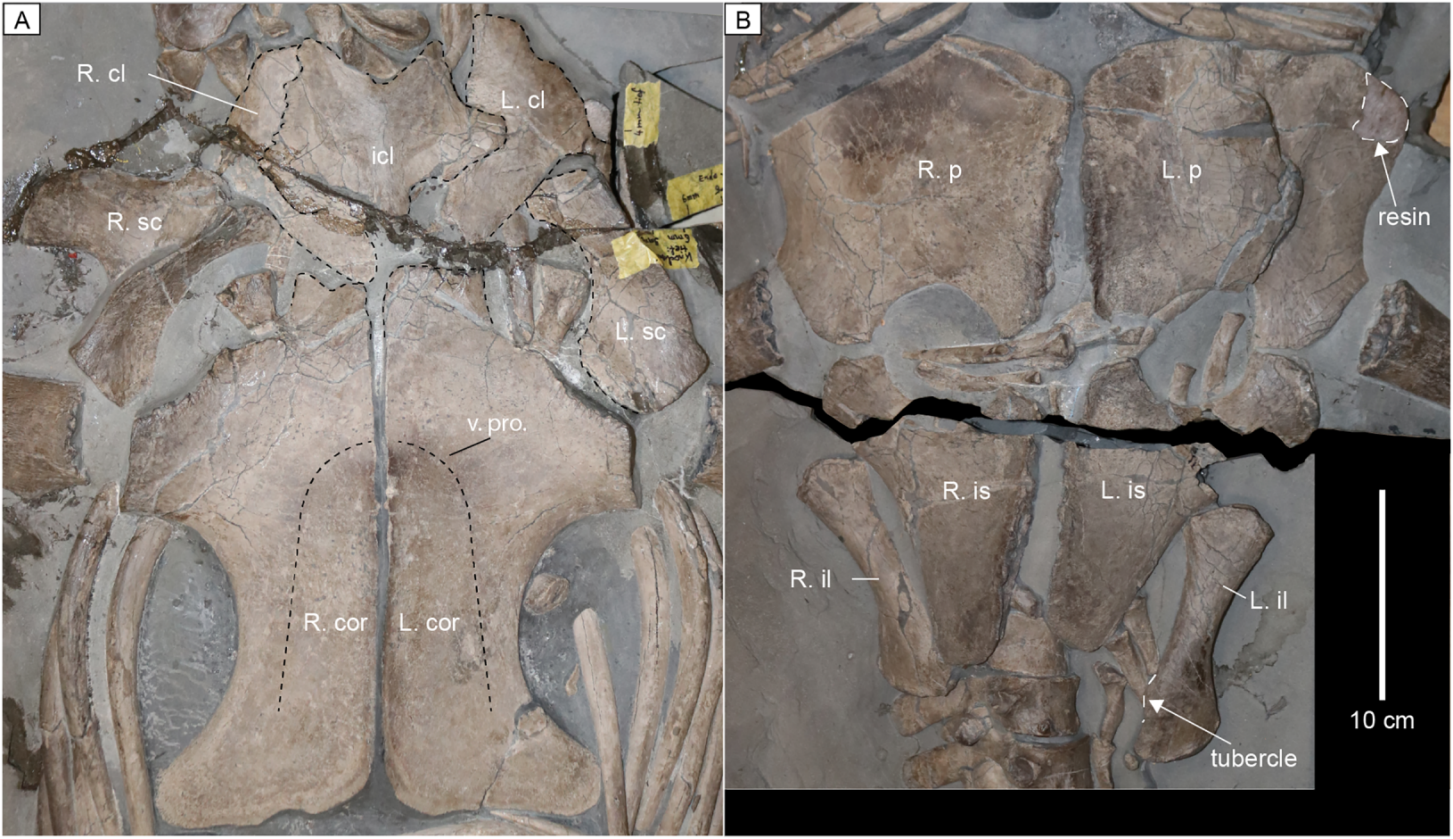
Pectoral and pelvic girdles of MH 7. (A) Pectoral girdle. (B) Pelvic girdle. Abbreviations: cl, clavicle; cor., coracoid; icl, interclavicle; il, ilium; is, ischium; p, pubis; sc, scapula; v. pro., ventral process. Note: the scale bar in panel B is also accurate for (A).

**Pectoral girdle.** The interclavicle is a broad star-shaped bone that forms the anteromedial margin of the pectoral girdle ventral to the clavicles (Fig. 10A). The anterior end of the interclavicle has a deep and broad U-shaped margin. Triangular anterolateral and lateral processes extend from the interclavicle. A short posterior extension appears to be present but is damaged.

Both clavicles have a triradiate outline in ventral view, with the posteromedial section contacting the anterior margin of the coracoids; this prevents the scapulae meeting along the midline, and from contacting the anteromedial processes of the coracoids. This interpretation is supported by the left scapula which remains in articulation with the clavicle and the coracoid (Fig. 10A).

The dorsal blade of the scapula curves posteriorly, terminating in a blunt apex that is approximately as wide as the body of the blade (Fig. 10A). The anterior margin of the dorsal blade is smoothly convex in outline, while the posterior margin of the blade is concave. On the lateral surface, the blade is flat with no process or elaborations. A distinct shelf on the ventrolateral margin is set at ~90° from the dorsal blade. The ventral surface of the scapula is smooth with a concave medial border. The facets for the coracoid and the humerus are approximately equal in width.

The coracoid forms a blunt and short anterior process (Fig. 10A). Laterally, the element widens to contact the scapula and receive the head of the humerus. A ventrally protruding eminence is noticeable along the center midline but flattens toward the glenoid and anterior process. The glenoid surface on the coracoid is a flat surface that forms a 90° angle with smoothly concave lateral margins posterior to the glenoid. The posterior processes of the coracoids extend as prominent cornua. See Table S2 for measurement data from the pectoral girdle.

**Pelvic girdle.** The pubis forms a smoothly convex anterior margin (Fig. 10B). The presence of an anterolateral cornu can be inferred in MH 7 as the lateral margin of the left pubis transitions from a concave surface to a convex one anteriorly that extends lateral to the acetabulum of the pelvic girdle; although the anterolateral corner of the left pubis is partly reconstructed, and the anterior margin of the right one is partly obscured. The medial surface is straight with the greatest thickness at mid-length. The surface of the acetabulum on the pubis is relatively long compared to that of the ischium. A posteromedial projection of the pubis completes a pelvic bar. The pelvic fenestra is well-defined and circular.

Along the anterior margin, the ischium forms a smoothly concave surface to contribute to the pelvic fenestra (Fig. 10B). The anteromedial process of the ischium is blunt where it contacts the pubis to form a pelvic bar. The medial surface of the ischium is nearly straight. The posterior process of the ischium is smoothly rounded. As in the pubis, the deepest region of the ischium is at approximately mid-length of the medial symphysis.

The proximal end of the ilium is double faceted to articulate with the ischium and contribute to the acetabulum. The ilium is nearly straight with only slight anterior curvature. There is no rotation of the distal end relative to the proximal end. A tubercule is located on the posterior surface. The exposed lateral, anterior, and posterior surfaces are otherwise smooth. The anterior face of the distal articular surface is more developed than the posterior articular end.

**Figure 11:**
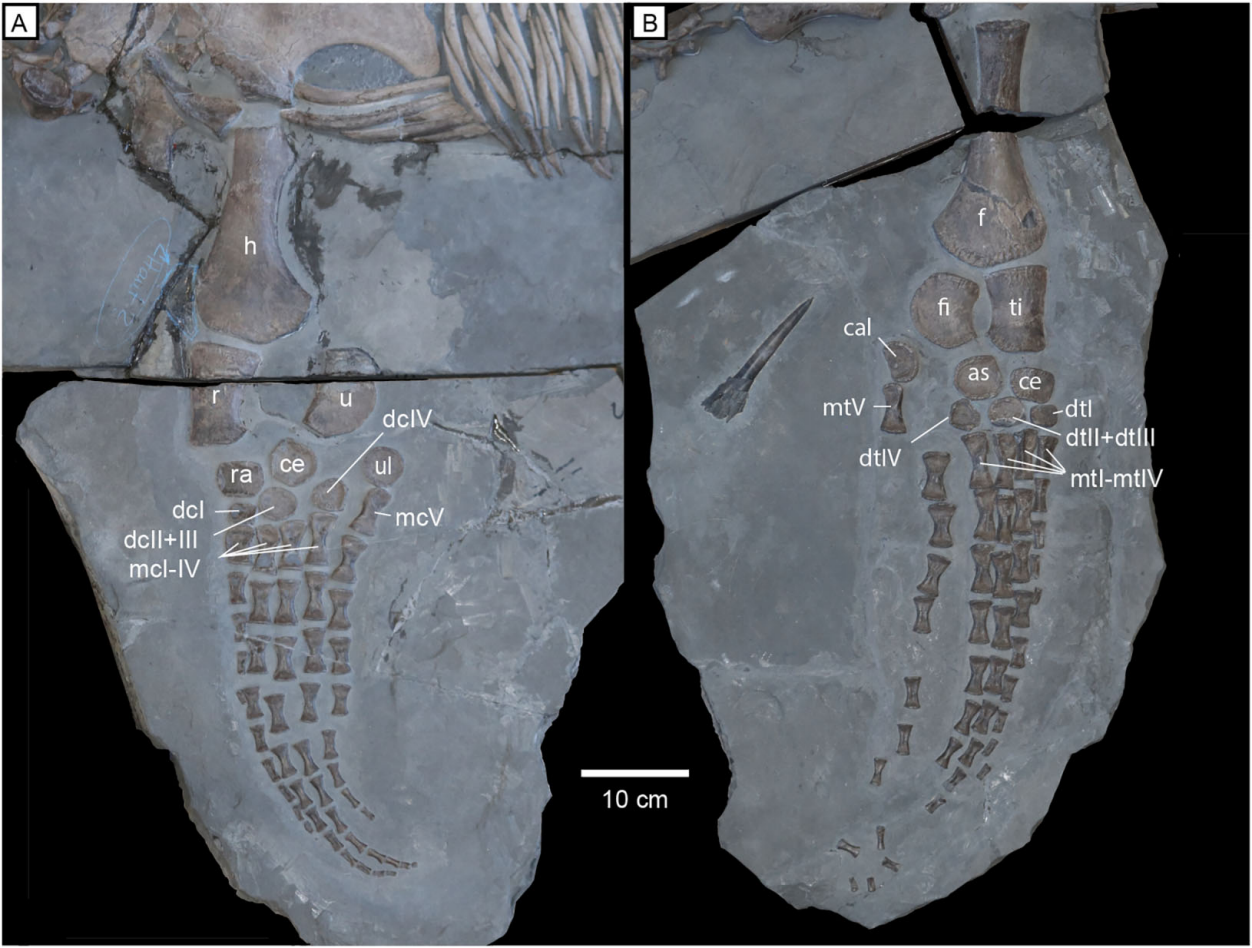
Limbs of MH 7. (A) Right forelimb. (B) Left hindlimb. Abbreviations: as, astragalus; cal, calcaneum; ce, centrale; dcI, distal carpal I; dcII+III, distal carpal II and III; dcIV, distal carpal IV; dtI, distal tarsal I; dtII+III, distal tarsal II+III; dtIV, distal tarsal IV; f, femur; fi, fibula; h, humerus; mcI-IV, metacarpals I through IV; mcV, metacarpal V; mtI-IV, metatarsal I-IV; mtV, metatarsal V; r, radius; ra, radiale; ti, tibia; u, ulna; ul, ulnare.

**Forelimb.** The humerus has a flat proximal head (Fig. 11A), which would have been covered by cartilage in life (Krahl, 2021). Posterior to the humeral head there is a protuberance that faces posteriorly. The posterior margin of the humerus is smoothly concave, and the anterior margin is almost straight. The distal end of the humerus has two distinct articular facets. A gentle posterodistal expansion can be observed while the anteriorodistal end of the humerus is only slightly expanded. The radius is columnar in overall shape with a flat proximal articular facet for the humerus and two distal facets, the larger being for the radiale and the smaller posterodistal facet for the intermedium. The anterior margin of the radius is shallowly concave, and the posterior margin is more deeply concave where it encloses the elongate epipodial foramen. The ulna is lunate in shape with one facet proximally and two distally to articulate with the centrale and the ulnare. Two rows of mesopodials are present in the forelimbs and hindlimbs of MH 7. There is no evidence of any pisiforms or accessory elements. The carpal bones are discoidal in shape, with distal carpal I being proximodistally compressed. The fifth metacarpal would have been situated posterior to distal carpal IV in the second row. The phalanges of the forelimb are spool-shaped and robust. The digit formula for the forelimb is 4-9-11-11-4. At the distal end of the second digit, a small round ossicle is present beyond the eighth digit. The distal end of both the forelimbs and the hindlimbs are strongly curved posteriorly.

**Hind limb.** The proximal end of the femur has a cratered surface and appears slightly rounder than the proximal head of the humerus (Fig. 11B). The pre- and postaxial margins of the femur are straight with a nearly symmetric distal expansion, as opposed to the humerus, which exhibits a greater posterodistal expansion. The distal width of the femur is approximately the same as in the humerus; however, the femur is shorter proximodistally (Table S3). The tibia is more robust than the radius in being proximodistally shorter with less curvature on the posterior margin, thus producing a squared profile. Likewise, the fibula is shorter and more robust in shape than the ulna.

No supernumerary elements are present in the fore or hind limbs. The tarsals are less disc-like than the carpals. The centrale is rectangular with a broad proximal facet for the tibia and the calcaneum is reniform with a slightly concave posterior margin. The astragalus has two distinct facets for articulation with the tibia and fibula. Distal tarsal I is rectangular in outline, and distal tarsal II + distal tarsal III is ellipsoidal. Distal tarsal IV presents a flat proximal surface for articulation with the astragalus.

The phalanges are all spool-shaped and robust with proximal widths equal to a little more than half the proximodistal length. In the more complete left hindlimb, four phalanges remain articulated in the first digit, eight in the second digit, eight in the third digit, six in the fourth digit, and four in the fifth digit. The exact digit formula of the hindlimb cannot be determined due to disarticulation of the phalanges at the distal end. However, based on the eight disarticulated phalanges distal to the left hind limb, the digit formula is likely 4-8-11-11-4, similar to that of the forelimb.

### Comparisons

The skull of MH 7 is disarticulated, with many of the cranial elements being obscured by matrix; however, some comparisons can be made with other Lower Jurassic plesiosaurians. For example, the ornamentation of the teeth in MH 7 is comparable to that of *Seeleyosaurus guilelmiimperatoris* (Maisch & Rücklin, 2000; Sachs et al., 2025), *Microcleidus melusinae* (Vincent et al., 2017b) and the holotype of *Plesiopterys wildi*, but different from that in *Microcleidus brachyptergius*, where small ridges are present along both the labial and lingual surface (Maisch & Rücklin, 2000). There are 16 dental alveoli in the maxilla of MH 7; that is, the same number as in *M*. *brachypterygius* (Maisch & Rücklin, 2000), but more than in *S*. *guilelmiimperatoris* (14 maxillary alveoli) (Maisch & Rücklin, 2000) and *Microcleidus tournemirensis* (12 maxillary alveoli) (Bardet, Godefroit & Sciau, 1999) and less than in *Plesiosaurus dolichodeirus* Conybeare, 1824, which has 18 maxillary teeth (Maisch & Rücklin, 2000).

The tall dorsal ramus of the maxilla at the anterior end of the orbit in MH 7 is distinct from the short and broader dorsal ramus of the maxilla in *M*. *brachypterygius* (Maisch & Rücklin, 2000), *M*. *melusinae* (Vincent et al., 2017b), *M*. *tournemirensis* (Bardet, Godefroit & Sciau, 1999), and *P*. *dolichodeirus* (Storrs, 1997). The holotype of *P*. *wildi* (SMNS 16812), however, also exhibits a narrow ramus at the anterior end of the maxilla where it forms the anterior margin of the orbit (Groβmann, 2007; fig. 4).

The short contribution to the ventral side of the temporal bar by the squamosal with a vertical, interdigitating suture where the jugal would have articulated in MH 7 (Fig. 4C) is distinct from that of *P*. *dolichodeirus* (Storrs, 1997), *M*. *tournemirensis*, *M*. *brachypterygius* (Maisch & Rücklin, 2000; Smith, Araújo & Mateus, 2011), and *Lusonectes sauvagei* Smith, Araújo & Mateus, 2011 which exhibit a longer contribution to the ventral side of the temporal bar by the squamosal. How the squamosal in MH 7 would have contributed to the dorsal margin of the temporal bar is, however, unknown as this portion of the skull is missing.

**Figure 12:**
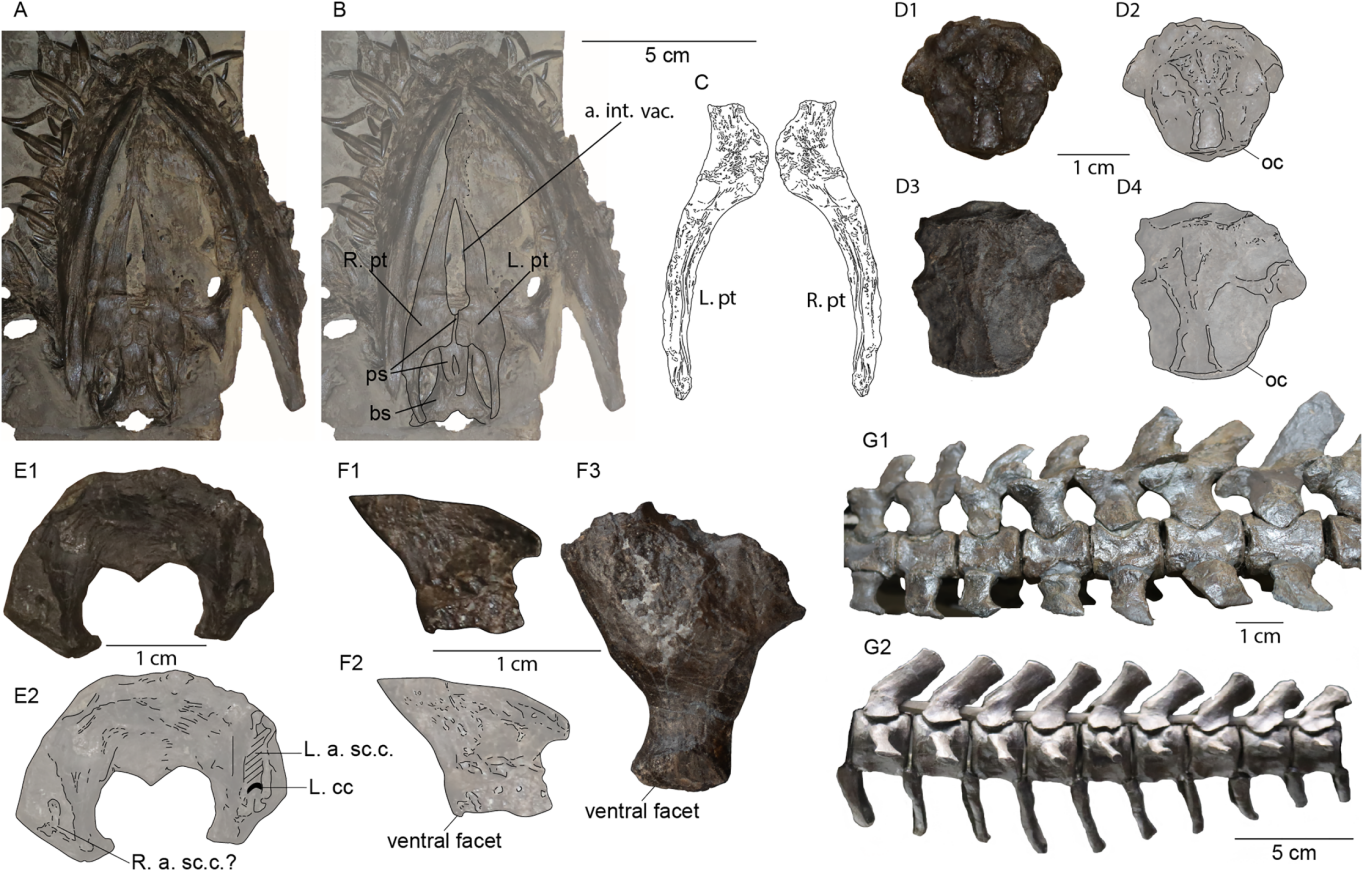
Comparisons of the holotype of *Plesiopterys wildi* (SMNS 16812) with MH 7. (A, B) Palate of *P. wildi* holotype (SMNS 16812) with right (R. pt) and left (L. pt) pterygoids labeled. (C) Right pterygoid of MH 7 in dorsal view (R. pt) with a symmetry of this element (L. pt) showing the partially preserved quadrate flanges of the pterygoids and medial flanges. (D1) Basioccipital of SMNS 16812 in dorsal view. (D2) Sketch of SMNS 16812 basioccipital. (D3) Basioccipital of MH 7 in dorsal view. (D4) Sketch of MH 7 basioccipital. (E1) Supraoccipital of SMNS 16812 in ventral view. (E2) Sketch of SMNS 16812 supraoccipital. (F1) Left lateral view of the left prootic from SMNS 16812. (F2) Sketch of prootic in SMNS 16812. (F3) Left lateral view of left prootic from MH 7. (G1) Anterior cervical series of SMNS 16812. (G2) Middle caudal vertebrae of SMNS 16812 with posteriorly reclining neural arches. Abbreviations: a. int. vac., anterior interpterygoid vacuity; a. sc.c, anterior semicircular canal; bs, basisphenoid; cc, crus communis; oc, occipital condyle; ps, parasphenoid; pt, pterygoid.

The shape of the mandible in MH 7 is similar to that of *M*. *melusinae* (Vincent et al., 2017b) and *P*. *dolichodeirus* (Storrs, 1997) with no apparent bowing. A ventral elaboration along the symphysis is also shared with *M*. *melusinae*, although the holotype of *P*. *wildi* (SMNS 16812) appears to lack this character, at least as reconstructed by Groβmann (2007, fig. 5) contra O’Keefe (2004). In our first-hand observation, we also find that *P*. *wildi* lacks this keel (Figs. 12A, 12B).

**Figure 13:**
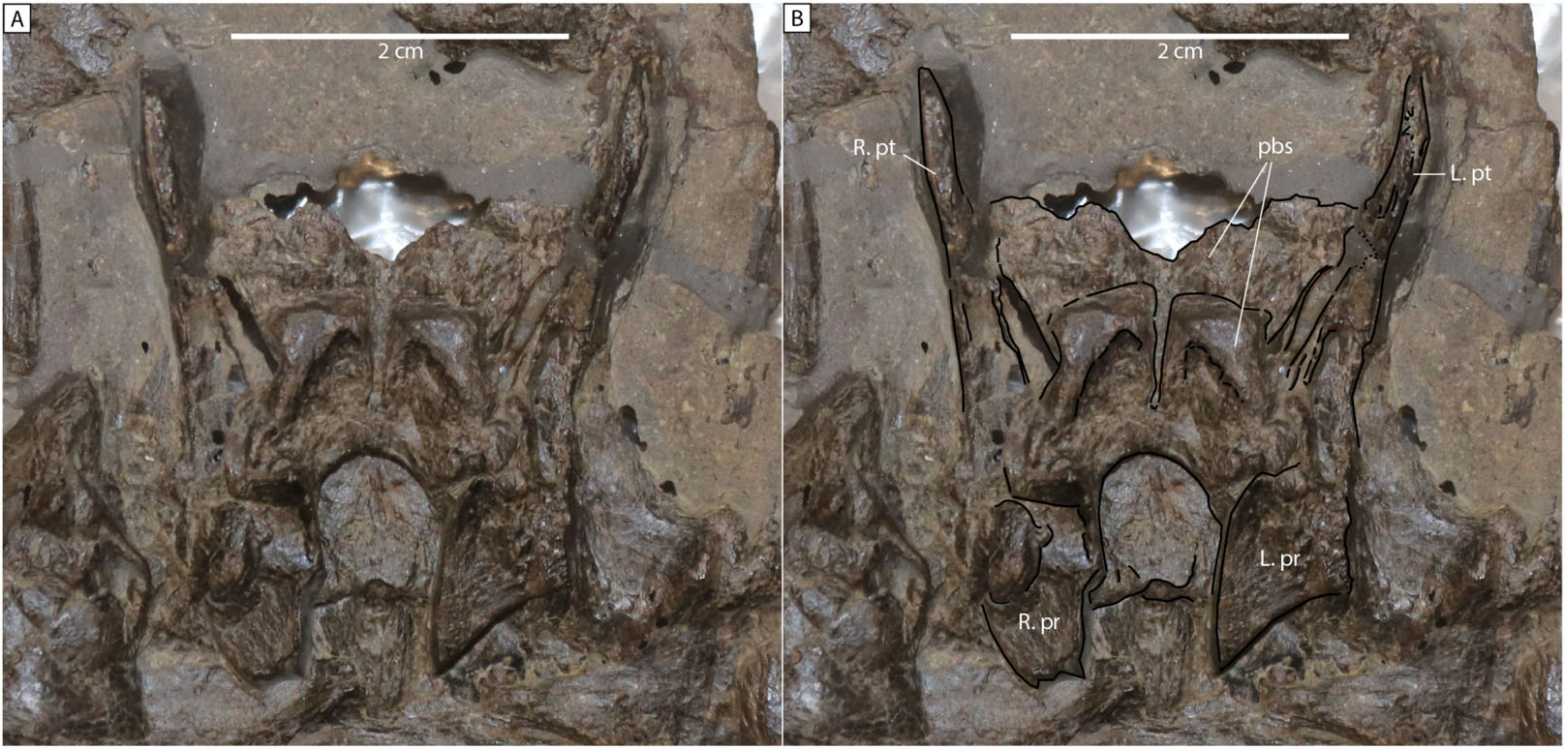
Dorsal view of the basicranium of SMNS 16812 (holotype of *Plesiopterys wildi*). (A) Dorsal view of the basicranial without labels. (B) Dorsal view of the basicranial with the pterygoids, parabasisphenoid, and prootics labeled. Abbreviations: pbs, parabasisphenoid; pr, prootic; pt, pterygoid.

With respect to the pterygoid in MH 7, this element is nearly identical to that of SMNS 16812 in several aspects. An elongated groove along the dorsal surface of the quadrate flange was regarded by O’Keefe (2004) as unique to *P*. *wildi*, however, close inspection reveals that the ‘groove’ in SMNS 16812 is sediment fill between the quadrate ramus of the pterygoid and the parabasisphenoid (Fig. 13). In the more mature MH 7, this groove on the pterygoid is apparent and has been previously regarded as a plesiomorphic condition (as it is present also in nothosaurids; Rieppel, 1994; Rieppel & Werneburg, 1998; O’Keefe, 2006).

The quadrate flange of the pterygoid in MH 7 is narrow and straight (Fig. 12C), as in SMNS 16812, but this character state is present also in *M*. *tournemirensis* (Bardet, Godefroit & Sciau, 1999) and *M*. *brachypterygius* (Maisch & Rücklin, 2000). However, in these latter two taxa, the pterygoid forms a sheet-like extension below the basicrania, which does not occur in SMNS 16812 or MH 7. The quadrate flange in MH 7 and SMNS 16812 does not develop a vertical flange, like in *M*. *melusinae* (Vincent et al., 2017b) and *M*. *tournemirensis* (Bardet, Godefroit & Sciau, 1999). O’Keefe (2004) interpreted the quadrate flange in SMNS 16812 as terminating in a boss; however, Groβmann (2007) reported this process as being broken. It is further worth noting that O’Keefe (2004) included the anterior interpterygoid vacuity with a rounded posterior margin and sharp anterior border as an autapomorphy for *P*. *wildi*. However, this character is also present in *Trinacromerum bentonianum* Cragin, 1888 (Williston, 1908; fig. 1) and possibly also *Tricleidus seeleyi* Andrews, 1910; (fig. 74). Furthermore, the autapomorphy defined by O’Keefe (2004, p. 973) for *P*. *wildi*: “exposure of the cultriform process of the parasphenoid almost to margin of anterior pterygoid vacuity” was reassessed and rather, the parasphenoid does extend to the margin of the anterior interpterygoid vacuity (Figs. 12A, 12B).

Anterior to the quadrate flange, a short and rounded medial flange from the pterygoid is apparent in MH 7, where the posterior interpterygoid vacuity would have terminated anteriorly (Fig. 12C). This is similar but more rectangular in outline in SMNS 16812 (Figs. 12A, 12B). In MH 7, the anterior portion of the pterygoid narrows rapidly following this medial flange. Thus, the anterior portion of the parasphenoid would likely have been close to the posterior opening of the anterior interpterygoid vacuity or contributed to its posterior border. The morphology of the pterygoid is different between MH 7 and SMNS 16812 with respect to the lateral margin, where the pterygoid of MH 7 is slightly concave as opposed to straight (Figs. 12B, 12C). These shared states of the palate are distinguishable from those of *M*. *brachypterygius*, *M*. *tournemirensis*, *P*. *dolichodeirus*, *M*. *melusinae*, *Microcleidus homalospondylus* (NHMUK 36184), and *L*. *sauvagei* which all lack the medial flange of the pterygoid separating the elongated interpterygoid vacuities (Smith, Araújo & Mateus, 2011; Vincent et al., 2017b). O’Keefe (2004) reported the medial extension of the pterygoid of SMNS 16812 as being dorsal to the plane of the palate. However, because the skull of SMNS 16812 is compressed, we consider this feature to be a taphonomic artefact.

In MH 7, the parabasisphenoid has a concave ventral surface (Fig. 4A), and thus is not flat as in *M*. *brachypterygius* (Maisch & Rücklin, 2000). No foramina are evident in ventral view, as opposed to the condition *M*. *tournemirensis* (Bardet, Godefroit & Sciau, 1999). The parasphenoid differs from that of SMNS 16812 in the lack of any processes or extensions on the ventral surface where O’Keefe (2004, p. 981) reports a “ventro-posterior process of the basisphenoid” similar to *Thalassiodracon hawkinsii* (Owen, 1838) and OUM J.28585, with the parasphenoid extending beyond this process to articulate with the quadrate flanges of the pterygoid and the basioccipital tuber (O’Keefe, 2004, fig. 3). We have reinterpreted the basicranium from that of O’Keefe (2004) (Figs. 12A, 12B, 13). However, the parabasisphenoid in *T*. *hawkinsii* apparently lacks this structure (Benson et al., 2011b). O’Keefe (2004) considered the extension of the parasphenoid beyond this process of the basispheniod to be unique to *P*. *wildi*. However, Groβmann (2007) did not interpret any process of the basisphenoid in *P*. *wildi*.

In view of the basioccipital, an eminence with a triangular outline tapers posteriorly along the dorsal surface. This same eminence can be found in SMNS 16812 (Figs. 12D1, D2); however, it is incipient and not as developed as in MH 7 (Figs. 12D3, 12D4). The basioccipital tubers are round and small processes, like those of SMNS 16812, but differing from those of *Stratesaurus taylori* Benson, Evans & Taylor, 2015 which are anteroposteriorly long. Similar to *M*. *tournemirensis*, there is no contribution to the occipital condyle by the exoccipital-opisthotics in MH 7.

O’Keefe (2004, fig. 5) interpreted the anterior semicircular canal as piercing the supraoccipital in SMNS 16812, similar to the condition in *T*. *hawkinsii* (Benson et al., 2011b), rather than an impression on the bone as in MH 7 and *S*. *taylori* (Benson, Evans & Taylor, 2015). We could not identify a foramen for the anterior semi-circular canal on the supraoccipital of SMNS 16812 as described by O’Keefe (2004). Rather, impressions in the bone for the anterior semicircular canal and the crus communis were apparent in the left-half of the supraoccipital (Figs. 12E1, 12E2). A smaller impression is apparent in the right-half and may represent an asymmetry in the contribution of the supraoccipital to the semicircular canals and crus communis is SMNS 16812 (Fig. 12E2). The endosseous labyrinth in MH 7 thus appears nearly identical to that of SMNS 16812.

The prootic is not often documented in Lower Jurassic plesiosaurians (Bardet, Godefroit & Sciau, 1999; Maisch & Rücklin, 2000; Smith & Benson, 2014; Vincent et al., 2017b). Yet, the prootic of MH 7 is very similar to that of *S*. *taylori* (Benson, Evans & Taylor, 2015, fig. 6D, E) but differs from that of *M*. *homalospondylus* (Brown, Vincent & Bardet, 2013, figs. 3.2, 3.6), which has an anteroventral process that Brown, Vincent & Bardet (2013) considered to be plesiomorphic among Plesiosauria. The prootic of SMNS 16812 also differs from MH 7 in possessing an elongate and flat dorsal margin without any obvious facets for the supraoccipital or exoccipital-opisthotic (Figs. 12F1–12F3). The ‘prootic’ bones described by Groβmann (2007) are isolated skeletal fragments located at the distal-most portion of the quadrate ramii of the pterygoids, and appear to belong to these elements, where they would have articulated with the quadrate. The medial face of the prootic in MH 7 is obscured by sediment; thus, the vestibule and foramina for cranial nerves cannot be described or compared (Fig. 12F3).

The hyoid of MH 7 exhibits an anterior end that is slightly greater in diameter than the posterior end, similar to the condition in *Meyerasaurus victor* and *S*. *taylori* (see Smith & Vincent, 2010; Benson, Evans & Taylor, 2015).

The estimated total cervical count for MH 7 (38) is higher than that of *M. melusinae* (32) (Vincent et al., 2017b), *S*. *guilelmiimperatoris* (34) (Dames, 1895; Sachs et al., 2025), but lower than that of *M. tournemirensis* (41) (Bardet, Godefroit & Sciau, 1999) and close to SMNS 16812 (39) (O’Keefe, 2004). The diapophyses and parapophyses on the posterior-most cervical vertebrae of MH 7 are not separated, unlike the condition in *Microcleidus* spp. (Benson, Evans & Druckenmiller, 2012). Nearly all the cervical vertebrae exhibit ribs that are hatchet-shaped with a more pronounced posterior process on the distal end and a reduced process anteriorly (Figs. 7A, 7B). This contrasts with the condition in *S*. *guilelmiimperatoris*, where significantly more pronounced anterior and posterior processes are present on the anterior cervical ribs (Dames, 1895); however, it is similar to *Franconiasaurus brevispinus* Sachs, Eggmaier & Madzia, 2024 and *Plesiopterys wildi* (Fig. 12G1). A keel on the lateral surface of the cervical vertebrae may be present in two anterior cervical vertebrae. However, unlike the condition in *M*. *homalospondylus* (Owen, 1865) and *M*. *tournemirensis* (Bardet, Godefroit & Sciau, 1999), this character is absent from the rest of the cervical column. Nevertheless, the presence of this character in MH 7 may be ontogenetically influenced. A lateral keel is not apparent in SMNS 16812 (Fig. 12G1). The shark fin-shaped anterior neural spines in MH 7 are similar to those of SMNS 16812 (Fig. 12G1) and *F*. *brevispinus* (Sachs, Eggmaier & Madzia, 2024) but unlike those of *S*. *guilelmiimperatoris* (Dames, 1895), which exhibits rectangular neural spines oriented straight dorsally. The presence of shark fin-shaped cervical neural spines in the osteologically immature holotype of *Brancasaurus brancai* Wegner, 1914 might suggest that this character is indicative of immature individuals (Sachs, Hornung & Kear, 2016). *M*. *melusinae* exhibits cervical neural spines inclined slightly anteriorly (Vincent et al., 2017b). The anterior cervical vertebrae of *M. tournemirensis* have a straight posterior margin that is slightly inclined posteriorly and a smoothly convex anterior margin (Bardet, Godefroit & Sciau, 1999). With respect to the caudal series, a minimum of 35 or 36 caudal vertebrae in MH 7 is greater than that of *M*. *brachypterygius* (28 caudal vertebrae in SMNS 51143, and MH 8), but less than SMNS 16812 (41) (O’Keefe, 2004). The posterior inflection of the caudal neural spines in the posterior half of the caudal series in MH 7 is identical to SMNS 16812 (Fig. 12G2).

**Figure 14:**
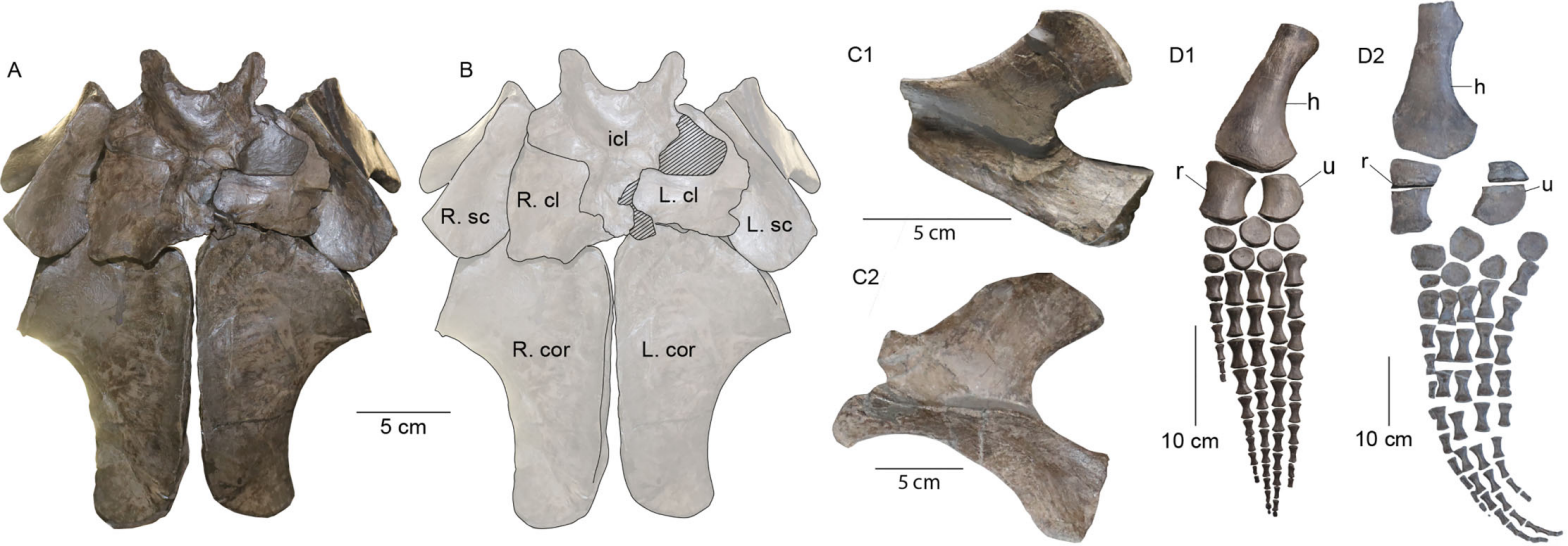
Girdle elements of SMNS 16812 (*Plesiopterys wildi* holotype) for comparison. (A) Pectoral girdle of SMNS 16812 in ventral view. (B) Sketch of the pectoral girdle of SMNS 16812. Cross-hatched areas are reconstructed areas/mount support. (C1) Left lateral view of the scapula from SMNS 16812. (C2) Lateral view of the right scapula from MH 7 (flipped asymmetrically to compare with SMNS 16812). (D1) Left front limb of SMNS 16812 in ventral view. (D2) Right front limb of MH 7 in ventral view. Abbreviations: cl, clavicle; cor, coracoid; h, humerus; icl, interclavicle; r, radius; sc, scapula; u, ulna.

The broad star-shaped interclavicle in MH 7 is similar to that of SMNS 16812 (Figs. 14A, 14B). *M*. *tournemirensis* (Bardet, Godefroit & Sciau, 1999), and *Westphaliasaurus simonsensii* Schwermann & Sander, 2011 exhibit interclavicles that are more funnel-shaped in outline. The scapulae in SMNS 16812 are similarly distanced from the anteromedial extensions of the coracoid. The clavicles of SMNS 16812 appear distinct from those of MH 7 in being crescent-shaped when viewed together ventrally, with a convex anterior margin and a more concave one posteriorly (Figs. 14A, 14B). The scapular blade of MH 7 is identical in morphology to that of *F*. *brevispinus* (Sachs, Eggmaier & Madzia, 2024) with a convex surface along its anterior margin, unlike that of SMNS 16812 which is more concave (Figs. 14C1, 14C2). The cornua in MH 7 do not extend laterally beyond the pectoral glenoid, similar to the condition in *M*. *tournemirensis* (Bardet, Godefroit & Sciau, 1999).

The anterior margin of the pubis in MH 7 is convex unlike that of *M. tournemirensis* (Bardet, Godefroit & Sciau, 1999) or *Plesiopharus moelensis* Puértolas-Pascual et al., 2021, which has a shallow excavation on the anterior border. The medial surface of the ischium is nearly straight, similar to that of *P*. *moelensis* (Puértolas-Pascual et al., 2021) but not *M. tournemirensis*, where the medial margins are smoothly curved (Bardet, Godefroit & Sciau, 1999).

With respect to the limbs, the radii of MH 7 lack the flange present on the proximal part of the radius in *Microcleidus* spp. and *S*. *guilelmiimperatorus* (Dames, 1895; Benson, Evans & Druckenmiller, 2012; Sachs et al., 2025). Similarly, SMNS 16812 lacks this flange (Figs. 14D1, 14D2). Additionally, MH 7 does not have supernumerary elements in either the fore—or hind limbs, as otherwise seen in *M*. *brachypterygius* (von Huene, 1923) and *S*. *guilelmiimperatoris* (Dames, 1895) but again, similar to SMNS 16812 (Figs. 14D1, 14D2).

### Phylogenetic analysis

**Figure 15:**
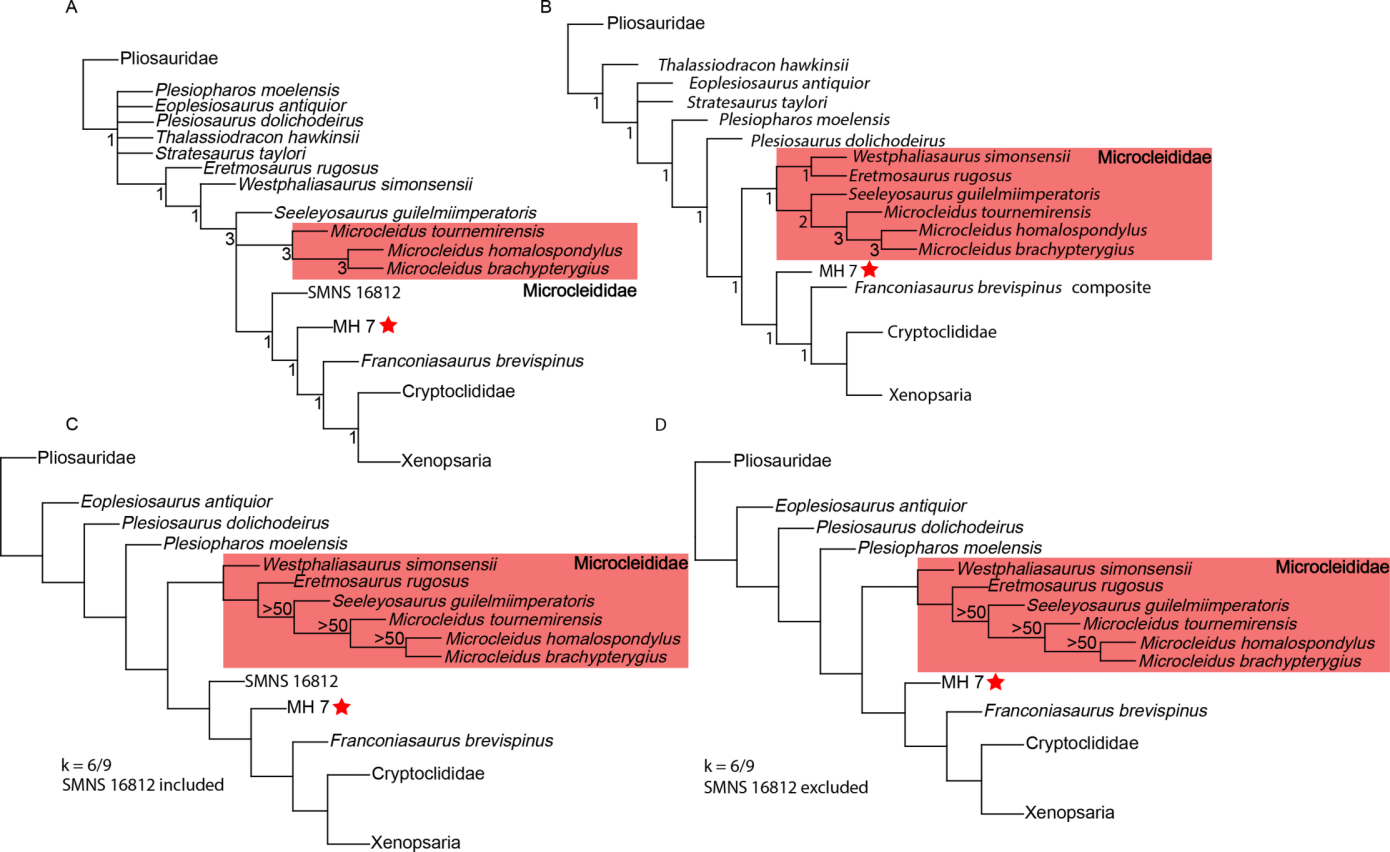
Phylogenetic analyses incorporating MH 7. (A) Phylogenetic placement of MH 7 from the ‘New Technology’ search with subsequent TBR. Bremer support indicated below nodes. (B) Phylogenetic placement of MH 7 excluding SMNS 16812 from the ‘New Technology’ search with subsequent TBR. Bremer support indicated below nodes. (C) Implied weighting analyses (k = 6/9) including SMNS 16812; symmetric resampling values above 50 below the nodes. (D) Implied weighting analyses (k = 6/9) excluding SMNS 16812; symmetric resampling values above 50 below the nodes.

We used similar analysis parameters to those of Sachs, Eggmaier & Madzia (2024) with an unweighted ‘New Technology’ search and two additional searches using implied weighting. These analyses were done with the *Plesiopterys wildi* holotype (SMNS 16812) and MH 7 included in the matrix, and a second round of analyses with the holotype excluded, for a total of six analyses. MH 7 was scored for 135 characters out of 270 used by Sachs, Eggmaier & Madzia (2024). Space for 200,000 trees was allocated to RAM (command: hold 200000;). Then, a ‘New Technology’ search with 1,000 addition sequences and default settings for tree fusing, ratchet, drift, and sectorial searches was used, which retained 41 trees (CI: 0.190; RI: 0.684; RCI: 0.130). A TBR search was then run on trees saved to RAM. This returned a best score of 2,098 with a consensus of Plesiosauroidea, including MH 7 in a more derived position than SMNS 16812, but basal to *Franconiasaurus brevispinus*. Together, SMNS 16812, MH 7 and *F. brevispinus* are reconstructed successively closer to cryptoclidian plesiosaurians; the placement of SMNS 16812 basal to MH 7 can be attributed to its juvenile ontogenetic stage. Bremer supports were calculated using the implemented script in TNT (“Trees” > “Bremer supports” > “Calculate Bremer supports” > “TBR from existing trees” > “Retain trees suboptimal by three steps”) (Fig. 15A). The second ‘New Technology’ search with SMNS 16812 removed retained 20 trees (CI: 0.191, RI: 0.683, RCI: 0.131; best score: 2,085). Subsequent TBR returned MH 7 again in an intermediate position between Microcleididae and *F*. *brevispinus* (Fig. 15B). This analysis recovered the following combination of character states diagnosing MH 7: morphology of ventral surface of the parasphenoid within the interpterygoid vacuity concave (ch.83: 2 -> 0), axial neural spine transversely broad (ch.150: 0 -> 1), chevron facets of middle and distal caudal vertebrae project significantly ventrally (ch.193: 0 -> 1), ratio of coracoid to scapula length is greater than or equal to 1.9 (ch.196: 1 -> 0), scapular dorsal process length relative to posterior ramus of the scapula is subequal or shorter (ch.204: 0 -> 1), ventral projection of the coracoid extends from the intercoracoid symphysis (ch.215: 0 -> 1).

Two implied weighting analyses were performed using two different concavity constants (k = 6 and 9). Space for 200,000 trees was again allocated and the same ‘New Technology’ search parameters were input for the implied weighting analyses. After the ‘New Technology’ search, the k = 6 weighted analysis retained 28 trees (CI: 0.188; RI: 0.680; RCI: 0.128), while the k = 9 analysis retained 13 trees (CI: 0.189; RI: 0.682; RCI: 0.129). After TBR swapping, the k = 6 analysis returned 32,319 trees, and the k = 9 analysis returned 161,595 trees. In both cases, MH 7 was returned as an early-diverging plesiosauroid and sister to *Franconiasaurus* + Cryptoclidia, with SMNS 16812 branching basally (Fig. 15C). Implied weighting (k = 6) without the holotype of *P*. *wildi* retained 15 trees (CI: 0.190, RI: 0.680, RCI: 0.129) with subsequent TBR swapping retaining 32,319 trees with MH 7 being intermediate again to *F*. *brevispinus* and more basal Lower Jurassic plesiosaurs, including Microcleididae. Implied weighting (k = 9) without the holotype retained 10 trees (CI: 0.190, RI: 0.681, RCI: 0.130) with subsequent TBR swapping resulting in 32,319 trees found with MH 7 being recovered in the same position as the k = 6 implied weighting analysis (Fig. 15D).

## Discussion

MH 7 shares two autapomorphic character states with the holotype of *Plesiopterys wildi* (SMNS 16812): medial flanges of the pterygoid separating the posterior interpterygoid vacuity from the anterior vacuity, and a star-shaped interclavicle with a deep anterior margin. Additional character states shared by SMNS 16812 and MH 7 include: cultriform process of the parasphenoid at the posterior margin of the anterior interpterygoid vacuity; possession of a narrow and straight quadrate ramus of the pterygoid; tall dorsal ramus of the maxilla; lack of supernumerary elements in the limb; lack of a proximal flange on the radius; and caudal neural spines of posterior caudal vertebrae inflected posteriorly. Given these similarities, we consider MH 7 to be osteologically more mature than SMNS 16812, and attribute all morphological differences to intraspecific and/or ontogenetic variation. The slight stratigraphic age difference between these two specimens is also minor, with MH 7 deriving from εII_6_ and SMNS 16812 from εII_4_.

Ontogenetically mature osteological character states apparent in MH 7 include: complete fusion of the neural arches to the centra; well-developed cornua on the coracoids; and well-defined facets on the propodial and epipodial elements (Brown, 1981; Araújo et al., 2015). However, MH 7 lacks fusion of the cervical and caudal ribs to the centra (see Brown, 1981). Additionally, the shark-fin shaped neural spines in MH 7, like those found in the osteologically immature holotype (GPMM A3.B4) of *Brancasaurus brancai* (Sachs, Hornung & Kear, 2016) and SMNS 16812 may be another juvenile character. This mixture of ‘adult’ and ‘juvenile’ character states implies a sub-adult ontogenetic stage for MH 7.

Groβmann’s (2007) assignment of SMNS 16812 as a juvenile *Seeleyosaurus guilelmiimperatoris* was based on shared character states of the dentition and craniofacial skeleton (Groβmann, 2007), in conjunction with a reinterpretation of the autapomorphic characters identified by O’Keefe (2004) for the pterygoid and palate. Groβmann (2007) reconstructed the medial extensions of the pterygoids anterior to the posterior interpterygoid vacuity as being the ventral surface of the parasphenoid (Groβmann, 2007, fig. 5). However, as we show, Groβmann (2007) reinterpretation of the pterygoid is incorrect. Although Groβmann (2007) was correct in dismissing the grooves O’Keefe (2004) reported on the quadrate process of the pterygoid in SMNS 16812. Yet, MH 7 does have elongated grooves on the dorsal surface of the pterygoids (Fig. 5A).

Character states from the dentition used to assign SMNS 16812 to *S*. *guilelmiimperatoris* are unreliable because the teeth cannot be accurately counted. Furthermore, the presence of striae (ridges) on the lingual side of the dentition in SMNS 16812 and lack of striae on the labial face is a common condition in plesiosaurians (Brown, 1981; Sachs et al., 2025) and therefore of poor taxonomic utility.

Additional ambiguous characters used by Groβmann (2007) to assign SMNS 16812 to *S*. *guilelmiimperatoris* include: (1) horizontally oriented and elongated rectangular jugal that forms part of the ventral orbit; (2) a maxillary-jugal suture with a right angle between its nearly vertical dorsal part and and horizontal ventral part; (3) squamosal with an anterodorsal process that contacts the postorbital and excludes the jugal from the temporal opening. For states (1) and (3), we could not substantiate these states in our assessment nor does Groβmann (2007; fig. 4, 5). Additionally, character (2) can be seen in other Early Jurassic plesiosaurs, such as M. brachypterygius (Maisch & Rücklin, 2000) and M. homalospondylus (Brown, Vincent & Bardet, 2013). The autapomorphies in SMNS 16812 shared with MH 7 thus establish *P*. *wildi* as a valid taxon. Furthermore, we recommend using MH 7 for *Plesiopterys wildi* in future phylogenetic analyses to avoid scoring of ontogenetically influenced states.

Our phylogenetic analyses recovered MH 7 as a derived Early Jurassic plesiosauroid more derived than Microcleididae. These results, along with the lack of widely separated rib facets of the posterior cervical vertebrae and a prominent flange extending anteriorly from the proximal portion of the radius (Benson, Evans & Druckenmiller, 2012), suggest a non-microcleidid affinity for MH 7 (*Plesiopterys wildi*). Derived cryptoclidian plesiosaurians appeared abruptly after the Toarcian (Fischer, Weis & Thuy, 2021). Our results in conjunction with those from Sachs, Eggmaier & Madzia (2024), reveal a stepwise evolution toward this derived clade and thus fills the gap for their appearance in the Early to Middle Jurassic.

Moreover, these results retain a high plesiosauroid diversity and possible endemism for the Toarcian Posidonienschiefer Formation of the Southwestern German Basin. It is of course possible that *P*. *wildi* could have dispersed into other basins in Central Europe (Sachs et al., 2016). However, the diverse plesiosaur fauna (at least five distinct genera and species) of the Southwestern German Basin (Maisch & Rücklin, 2000; O’Keefe, 2001; Smith & Vincent, 2010; Sachs et al., 2025) are distinct from that of the nearby Yorkshire Basin. With that being said, it is worth pointing out that the intraspecific variability among plesiosaurs within these basins remains understudied; some taxonomically segregated species of plesiosaurs may in fact be different ontogenetic stages of the same species or reflect sexual dimorphism. Nonetheless, the endemism of these Toarcian plesiosaurs is at odds with coeval ichthyosaurs (Fischer, Guiomar & Godefroit, 2011) and actinopterygians (Wretman, Blom & Kear, 2016), which were more dispersed in the European Epicontinental Sea. Perhaps plesiosaurs were more ecologically specialized than previously recognized and thus restricted to local areas. The Yorkshire Basin for example includes large-bodied macro-predatory rhomaleosaurids (Taylor, 1992), such as *Rhomaleosaurus thorntoni*, *Rhomaleosaurus cramptoni*, and *Rhomaleosaurus* *zetlandicus*, which reached sizes greater than five meters (Smith & Dyke, 2008). By comparison, the rhomaleosaurid *Meyerasaurus victor*, in the Southwestern German Basin, only reached 3.35 m in length (Smith & Vincent, 2010; fig. 1). *P*. *wildi*, *S*. *guilelmiimperatoris*, *Microcleidus brachypterygius*, and *Hauffiosaurus zanoni* similarly reached lengths of between 3 and 3.5 m (Groβmann, 2007; Vincent, 2011; Sachs et al., 2025). Perhaps niche separation can explain this disparity, with rhomaleosaurids in the more northern Yorkshire Basin possibly feeding on larger fish and cephalopods (Taylor, 1992; Sachs et al., 2023), while plesiosaurs in the Southwestern German Basin preyed on smaller-sized food items.

## Conclusions

MH 7 is a new plesiosaur from Holzmaden, represented by an articulated skeleton. MH 7 represent a sub-adult individual of *Plesiopterys wildi*. Shared autapomorphies between MH 7 and the holotype (SMNS 16812) demonstrate that *P*. *wildi* is a valid taxon and not synonymous with *Seeleyosaurus* or *Microcleidus*. This reinforces the high diversity of plesiosaurs in the Southwestern German Basin and its unique assemblage from coeval localities in the Yorkshire Basin of England. Our results thus infer possible endemism and regionalization of plesiosaur taxa within the Central European Basin during the Early Jurassic. Lastly, MH 7 is a derived non-microcleidid plesiosaur that together with *Franconiasaurus brevispinus*, form a stepwise evolution toward derived cryptoclidids. The Early Jurassic was therefore a critical interval for not only the early radiation of plesiosaurs but also the establishment of precursors to more derived forms that dominated the Late Jurassic.

## Supplemental Information

10.7717/peerj.18960/supp-1 Supplemental Information 1 Visual guide for lines of measurement from the appendicular skeleton of MH 7 used for supplementary tables 2 & 3. (A) Proximal portion of left forelimb with length of the left humerus added together in two segments (due to breakage) and distal width indicated. The length and widths of the left radius and ulna are also indicated. (B) Length and width of the right humerus. (C) Length and width measurements of the right radius and ulna. (D) Length of the better exposed left coracoid, widths of the coracoids, and lengths of the scapulae. (E) Length of the left femur added together in two segments and distal width indicated. The length and widths of the left tibia and fibula are also shown. (F) Length of the right femur added together in two segments and distal width shown. The proximal width of the right tibia is also annotated. (G) Length and minimum width of the left pubis along with the length (measured in two parts) and width of the left ischium. The proximal width of the left ilium along with its length is also indicated.

10.7717/peerj.18960/supp-2 Supplemental Information 2 Supplementary Tables documenting measurement data from the vertebrae and appendicular skeleton of MH 7.

10.7717/peerj.18960/supp-3 Supplemental Information 3 Character changes for *Plesiopterys wildi* holotype: SMNS 16812. Eight character changes were made to SMNS 16812:
Character 55: state 0 changed to 1; temporal bar is weakly embayed, or not embayed, temporal bar does not significantly arch dorsally.
Character 83: state 1 changed to 0; ventral surface of parasphenoid within the interpterygoid vacuity is mediolaterally concave.
Character 151: ? changed to 2; length to height ratio of the atlas-axis complex is at least 1.5 times that of the height.
Character 153: state 2 changed to 1; proportions of anterior to middle cervical vertebrae are approximately as long as high. State 2 is present in some elasmosaurid plesiosaurians.
Character 192: state 0 changed to [01]; the chevron facet of the caudal vertebrae is located primarily on both the anterior and posterior faces of some caudal centra, and primarily on the posterior face of other caudal centra.
Character 219: state 3 changed to 0; the anterior margin of the clavicle/interclavicle complex is deeply concave with a width at least 1.25 times the anteroposterior depth.
Character 257: state 1 to 0; a prominent anterior flange noes not extend from the anteroproximal face of the radius.
Character 260: state 1 to 0; expansion of the distal end of the ulna relative to the shaft is absent or very weak.

10.7717/peerj.18960/supp-4 Supplemental Information 4 Complete TNT matrix used to run phylogenetic analyses. The complete matrix that contains 270 characters and 131 OTUs. Modified by eight character changes to SMNS 16812 (*Plesiopterys wildi* holotype) and the addition of MH 7 from Sachs, Eggmaier & Madzia (2024).

10.7717/peerj.18960/supp-5 Supplemental Information 5 Complete list of all consensus trees from our phylogenetic analyses. All of our consensus trees from our six phylogenetic analyses (both weighted and unweighted) with bemer indices and symmetric resampling values reported at the nodes.

10.7717/peerj.18960/supp-6 Supplemental Information 6 All of our trees saved after the ’New Technology’ search and TBR from our unweighted parsimony analysis of 131 OTUs (includes SMNS 16812).

## Institutional abbreviations

GPIH: Institut für Geologie, Universität Hamburg, Hamburg, Germany
GPMM: Geomuseum der Universität Münster, Münster in Westfalen, Germany
MH: Urwelt-Museum Hauff, Holzmaden, Germany
NHMUK: Natural History Museum, London, United Kingdom
OUM: Oxford University Museum of Natural History, Oxford, United Kingdom
SMNS: Staatliches Museum für Naturkunde Stuttgart, Stuttgart, Germany

## Additional Information and Declarations

### Data Availability

The following information was supplied regarding data availability:

The matrix with taxon names and morphological character scores, along with our trees from one of our consensus analyses analyzing all 131 OTUs, are available in the Supplemental Files.
