# Supplementary figures and images for: A new specimen of Plesiopterys wildi reveals the diversification of cryptoclidian precursors and possible endemism within European Early Jurassic plesiosaur assemblages
Source: PeerJ. 2025 Mar 31;13:e18960. doi: 10.7717/peerj.18960 (PMC11967415; doi:10.7717/peerj.18960)

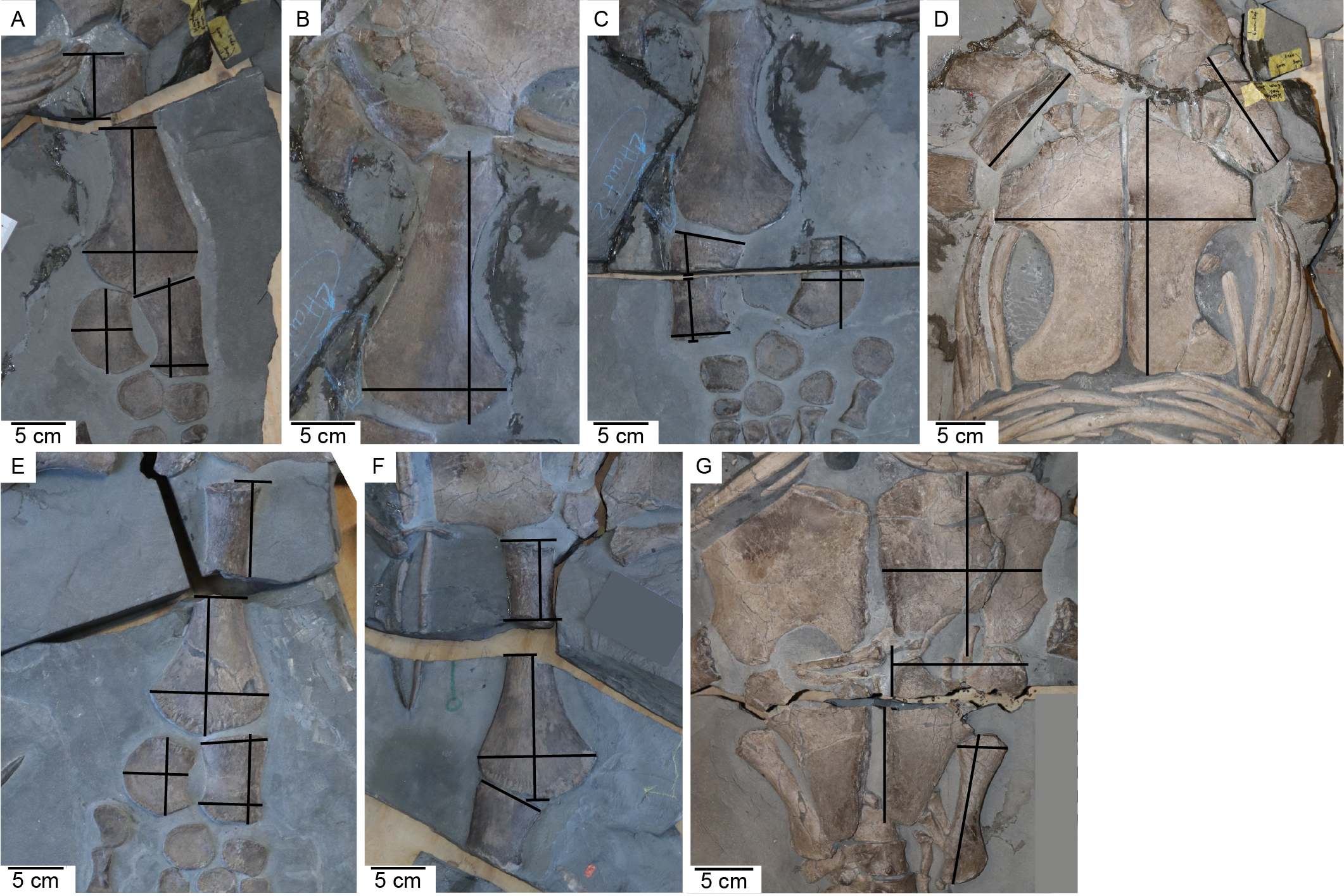

Supplement: Supplemental Information 1 — (A) Proximal portion of left forelimb with length of the left humerus added together in two segments (due to breakage) and distal width indicated. The length and widths of the left radius and ulna are also indicated. (B) Length and width of the right humerus. (C) Length and width measurements of the right radius and ulna. (D) Length of the better exposed left coracoid, widths of the coracoids, and lengths of the scapulae. (E) Length of the left femur added together in two segments and distal width indicated. The length and widths of the left tibia and fibula are also shown. (F) Length of the right femur added together in two segments and distal width shown. The proximal width of the right tibia is also annotated. (G) Length and minimum width of the left pubis along with the length (measured in two parts) and width of the left ischium. The proximal width of the left ilium along with its length is also indicated. [file peerj-13-18960-s001.png]
