# Supplementary material for: A new specimen of Plesiopterys wildi reveals the diversification of cryptoclidian precursors and possible endemism within European Early Jurassic plesiosaur assemblages
Source: PeerJ. 2025 Mar 31;13:e18960. doi: 10.7717/peerj.18960 (PMC11967415; doi:10.7717/peerj.18960)
Supplement: Supplemental Information 2 [file peerj-13-18960-s002.docx]

Supplementary Table 1. Measurement data from the cervical and caudal series of MH 7. Length was measured along the exposed lateral surface of the centra, or as otherwise noted, along the ventral surface. The length of three caudal vertebrae were measured along their exposed ventrolateral surface. Height was mainly measured using the anterior articular facet in lateral view, with the posterior facet used when the anterior one was obscured. Width was measured using the anterior articular facet unless otherwise stated as being measured from the posterior articular facet (post. facet). Measurements relied upon photographs with a scale bar and caliper measurements rounded to the nearest millimeter and thus these measurements should be taken as approximate values. The caudal vertebrae could be measured up to caudal vertebra 25.

| **Vertebra series** | **Length** | **Height** | **Width** |
| --- | --- | --- | --- |
| Atlas-axis complex | 28 mm | 15 mm | NA |
| Cervical 3 | NA | 15 mm (post. facet) | NA |
| Cervical 4 | 16 mm | NA | 19 mm |
| Cervical 5 | 17 mm | NA | NA |
| Cervical 6 | 18 mm | 18 mm | NA |
| Cervical 7 | 19 mm | 18 mm | NA |
| Cervical 8 | 19 mm | 19 mm | NA |
| Cervical 9 | 21 mm | 18 mm | NA |
| Cervical 10 | 22 mm | 19 mm | NA |
| Cervical 11 | 23 mm | NA | NA |
| Cervical 12 | 24 mm | NA | NA |
| Cervical 13 | 25 mm | 20 mm | NA |
| Cervical 14 | 25 mm | 23 mm | NA |
| Cervical 15 | 27 mm | 23 mm | NA |
| Cervical 16 | 27 mm | NA | NA |
| Cervical 17 | 28 mm (ventrally) | NA | NA |
| Cervical 18 | 29 mm | NA | NA |
| Cervical 19 | 30 mm | 28 mm | NA |
| Cervical 20 | 31 mm | 28 mm | NA |
| Cervical 21 | 31 mm | 28 mm | NA |
| Cervical 22 | 32 mm | 27 mm (post. facet) | NA |
| Cervical 23 | 32 mm | 27 mm | NA |
| Cervical 24 | 33 mm | 30 mm | NA |
| Cervical 25 | 33 mm | 28 mm | NA |
| Cervical 26 | 34 mm | NA | NA |
| Cervical 27 | 34 mm | NA | NA |
| Cervical 28 | 36 mm | NA | NA |
| Cervical 29 | 36 mm | NA | NA |
| Cervical 30 | 35 mm | NA | NA |
| Cervical 31 | 36 mm | NA | NA |
| Cervical 32 | 37 mm | NA | NA |
| Cervical 33 | 36 mm | NA | NA |
| Cervical 34 | 36 mm | NA | NA |
| Cervical 35 | 35 mm | NA | 43 mm |
| Cervical 36 | 34 mm (ventrally) | NA | 44 mm |
| Cervical 37 | 34 mm (ventrally) | NA | 45 mm |
| Cervical 38 | 33 mm (ventrally) | NA | 47 mm |
| Caudal 1 | 28 mm | 33 mm | NA |
| Caudal 2 | 28 mm | 30 mm | NA |
| Caudal 3 | 26 mm | 31 mm | NA |
| Caudal 4 | 26 mm | 32 mm | NA |
| Caudal 5 | 26 mm | 31 mm | NA |
| Caudal 6 | 26 mm | NA | NA |
| Caudal 7 | 26 mm | NA | NA |
| Caudal 8 | 25 mm | NA | NA |
| Caudal 9 | 25 mm | NA | NA |
| Caudal 10 | 25 mm | NA | NA |
| Caudal 11 | 23 mm | NA | NA |
| Caudal 12 | 24 mm | NA | NA |
| Caudal 13 | 21 mm | NA | NA |
| Caudal 14 | 21 mm (ventrolaterally) | NA | NA |
| Caudal 15 | 20 mm (ventrolaterally) | NA | NA |
| Caudal 16 | 21 mm (ventrolaterally) | NA | NA |
| Caudal 17 | 22 mm (ventrally) | NA | NA |
| Caudal 18 | 21 mm (ventrally) | 27 mm (post. facet) | 32 mm (post. facet) |
| Caudal 19 | 21 mm (ventrally) | NA | NA |
| Caudal 20 | 20 mm (ventrally) | NA | NA |
| Caudal 21 | 21 mm (ventrally) | NA | 25 mm (post. facet) |
| Caudal 22 | 21 mm (ventrally) | NA | 23 mm |
| Caudal 23 | 18 mm | 20 mm | NA |
| Caudal 24 | 18 mm | 19 mm | NA |
| Caudal 25 | 16 mm | NA | NA |

Supplementary Table 2. Measurement data from the girdle elements (excluding limbs) of MH 7. Measurements relied upon photographs with a scale bar rounded to the nearest tenth of a centimeter and thus these measurements should be taken as approximate values. See Supplementary Figure 1 for our measurement guidance.

| Length of the left coracoid: | 28.0 cm | Length of the left pubis: | 15.8 cm | Ilium proximal width: | 4.3 cm |
| --- | --- | --- | --- | --- | --- |
| Maximum width of the coracoids: | 26.4 cm | Width of the left pubis at approximately mid-length: | 13.7 cm | Ilium length: | 13 cm |
| Length of right scapula: | 12.4 cm | Left ischium anteroposterior length: | 14.3 cm |  |  |
| Length of left scapula: | 12.8 cm | Left ischium width: | 11.7 cm |  |  |

Supplementary Table 3. Measurement data from the limbs of MH 7. Measurements relied upon photographs with a scale bar rounded to the nearest tenth of a centimeter and thus these measurements should be taken as approximate values. Lengths of propodials, radii, ulnae, tibiae, and fibulae are measured proximodistally. See Supplementary Figure 1 for our measurement guidance.

| length of left humerus: | 21.9 cm | length of right humerus: | 21.4 cm | length of left femur: | 21.5 cm | length of right femur: | 21.3 cm |
| --- | --- | --- | --- | --- | --- | --- | --- |
| Distal width of left humerus: | 10.9 cm | Distal width of right humerus: | 11.3 cm | Distal width of left femur: | 10.9 cm | Distal width of right femur: | 11.3 cm |
| length of left radius: | 9.4 cm | length of right radius: | 9.8 cm | length of left tibia: | 8.3 cm | Proximal facet of right tibia: | 6.3 cm |
| Width of proximal facet from left radius: | 6.1 cm | Width of proximal facet from right radius: | 6.7 cm | Proximal facet of left tibia: | 6.1 cm |  |  |
| Distal width of left radius: | 5.5 cm | Distal width of right radius: | 5.6 cm | Maximum distal width of left tibia: | 5.9 cm |  |  |
| length of left ulna: | 8.1 cm | length of right ulna: | 8 cm | length of left fibula: | 7.2 cm |  |  |
| Maximum width of left ulna: | 5.8 cm | Maximum width of right ulna: | 5.8 cm | Maximum width of left fibula at mid-length: | 6 cm |  |  |
