# Supplementary material for: A new specimen of Plesiopterys wildi reveals the diversification of cryptoclidian precursors and possible endemism within European Early Jurassic plesiosaur assemblages
Source: PeerJ. 2025 Mar 31;13:e18960. doi: 10.7717/peerj.18960 (PMC11967415; doi:10.7717/peerj.18960)
Supplement: Supplemental Information 3 — Eight character changes were made to SMNS 16812: Character 55: state 0 changed to 1; temporal bar is weakly embayed, or not embayed, temporal bar does not significantly arch dorsally. Character 83: state 1 changed to 0; ventral surface of parasphenoid within the interpterygoid vacuity is mediolaterally concave. Character 151: ? changed to 2; length to height ratio of the atlas-axis complex is at least 1.5 times that of the height. Character 153: state 2 changed to 1; proportions of anterior to middle cervical vertebrae are approximately as long as high. State 2 is present in some elasmosaurid plesiosaurians. Character 192: state 0 changed to [01]; the chevron facet of the caudal vertebrae is located primarily on both the anterior and posterior faces of some caudal centra, and primarily on the posterior face of other caudal centra. Character 219: state 3 changed to 0; the anterior margin of the clavicle/interclavicle complex is deeply concave with a width at least 1.25 times the anteroposterior depth. Character 257: state 1 to 0; a prominent anterior flange noes not extend from the anteroproximal face of the radius. Character 260: state 1 to 0; expansion of the distal end of the ulna relative to the shaft is absent or very weak. [file peerj-13-18960-s003.docx]

**Character changes for *Plesiopterys wildi* holotype: SMNS 16812**


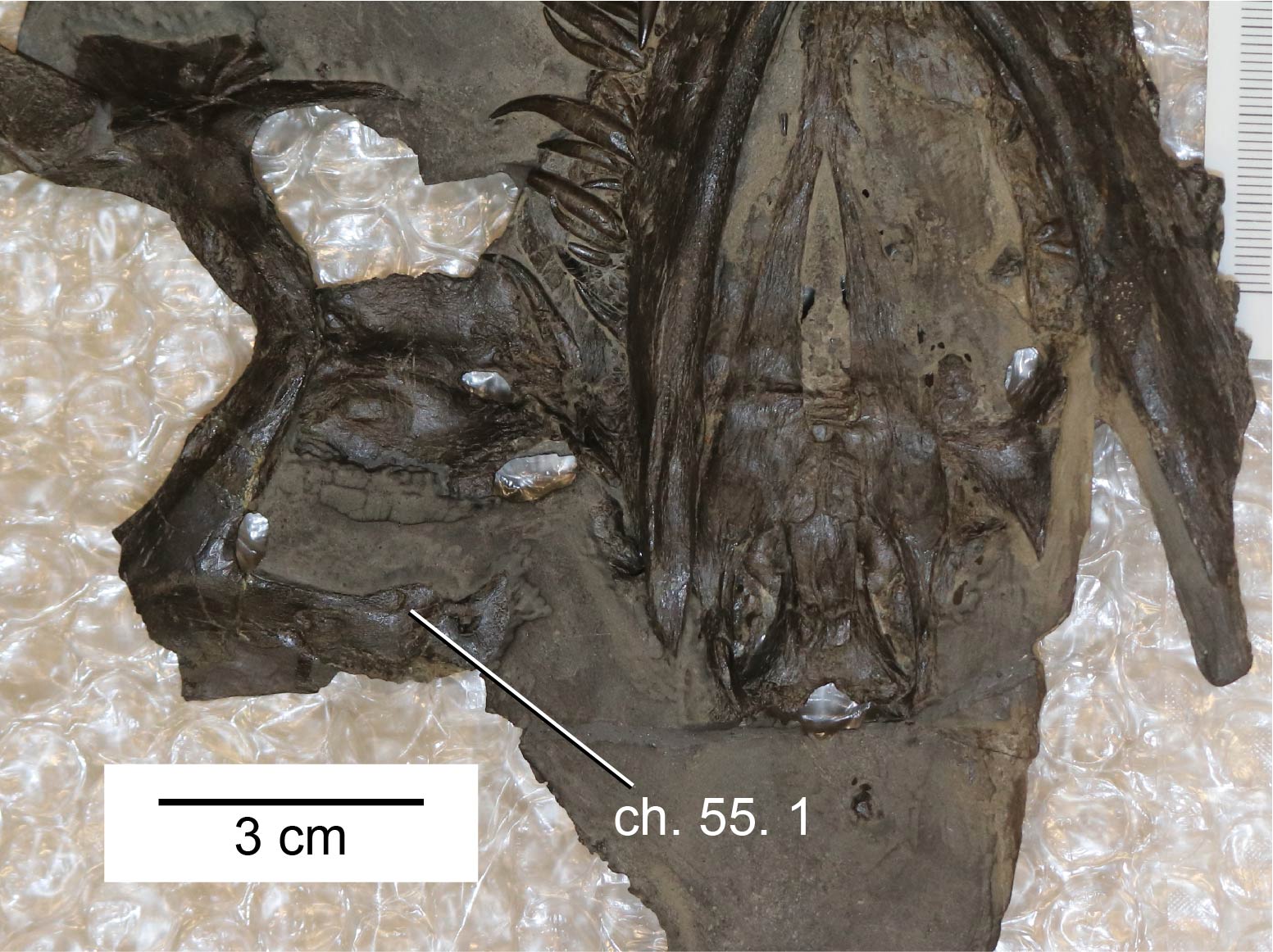


Character 55: state 0 changed to 1; temporal bar is weakly embayed, or not embayed, temporal bar does not significantly arch dorsally.


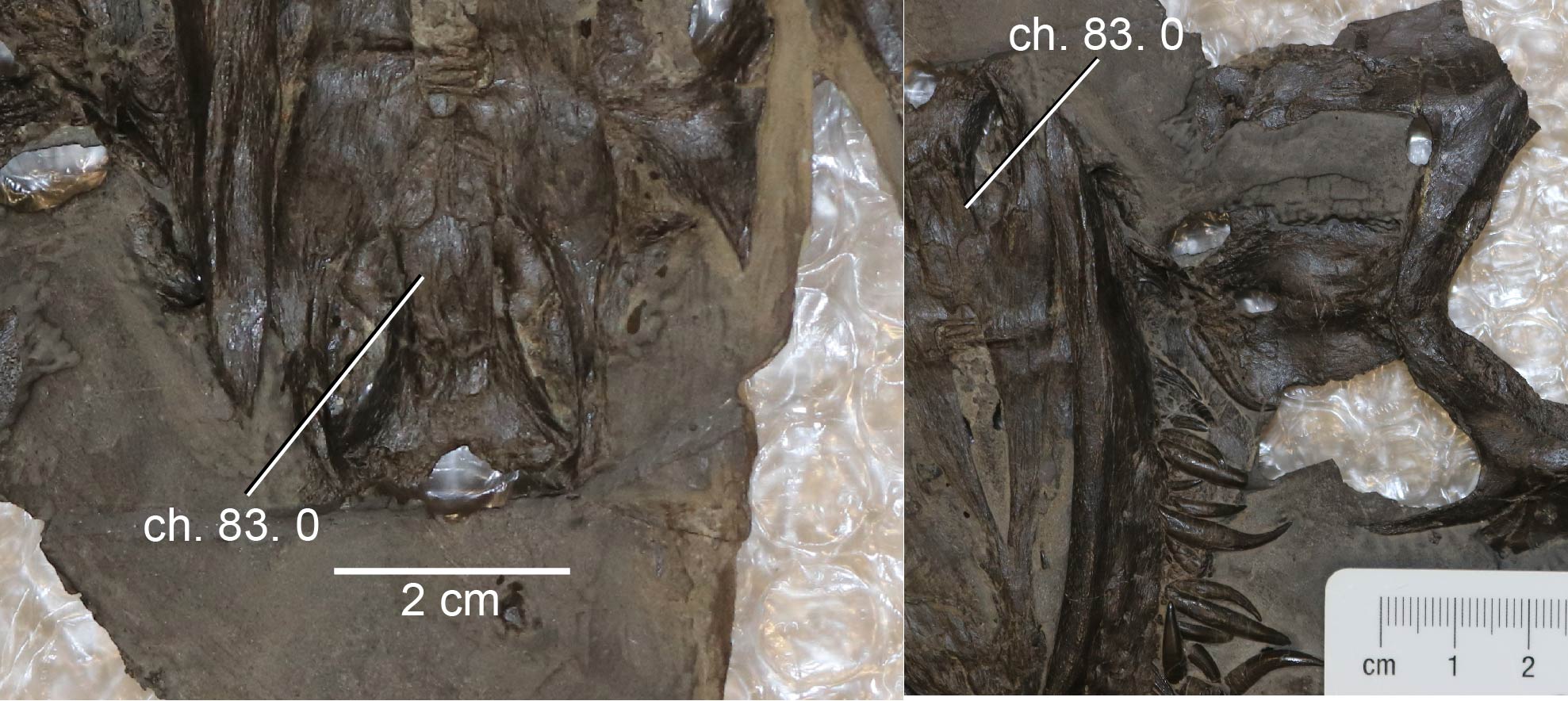


Character 83: state 1 changed to 0; ventral surface of parasphenoid within the interpterygoid vacuity is mediolaterally concave


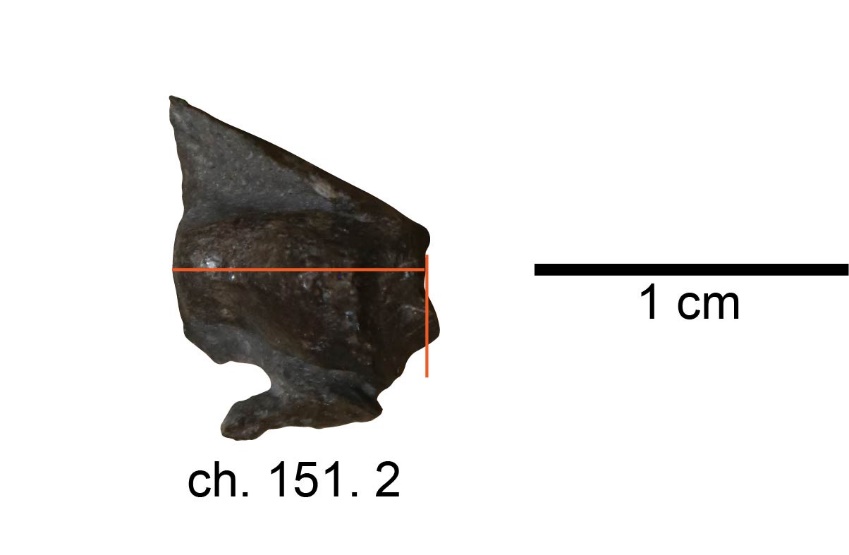


Character 151: ? changed to 2; length to height ratio of the atlas-axis complex is at least 1.5 times that of the height.


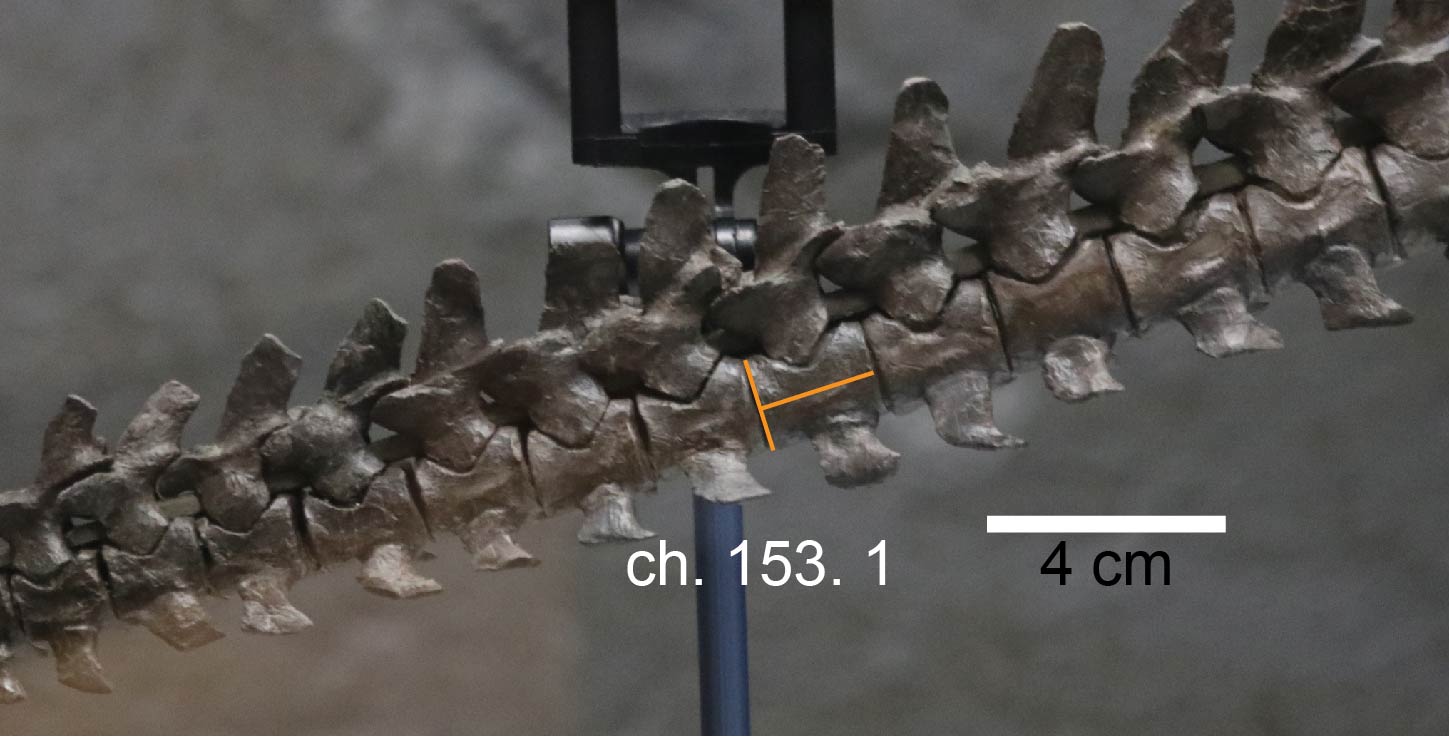


Character 153: state 2 changed to 1; proportions of anterior to middle cervical vertebrae are approximately as long as high. State 2 is present in some elasmosaurid plesiosaurians.


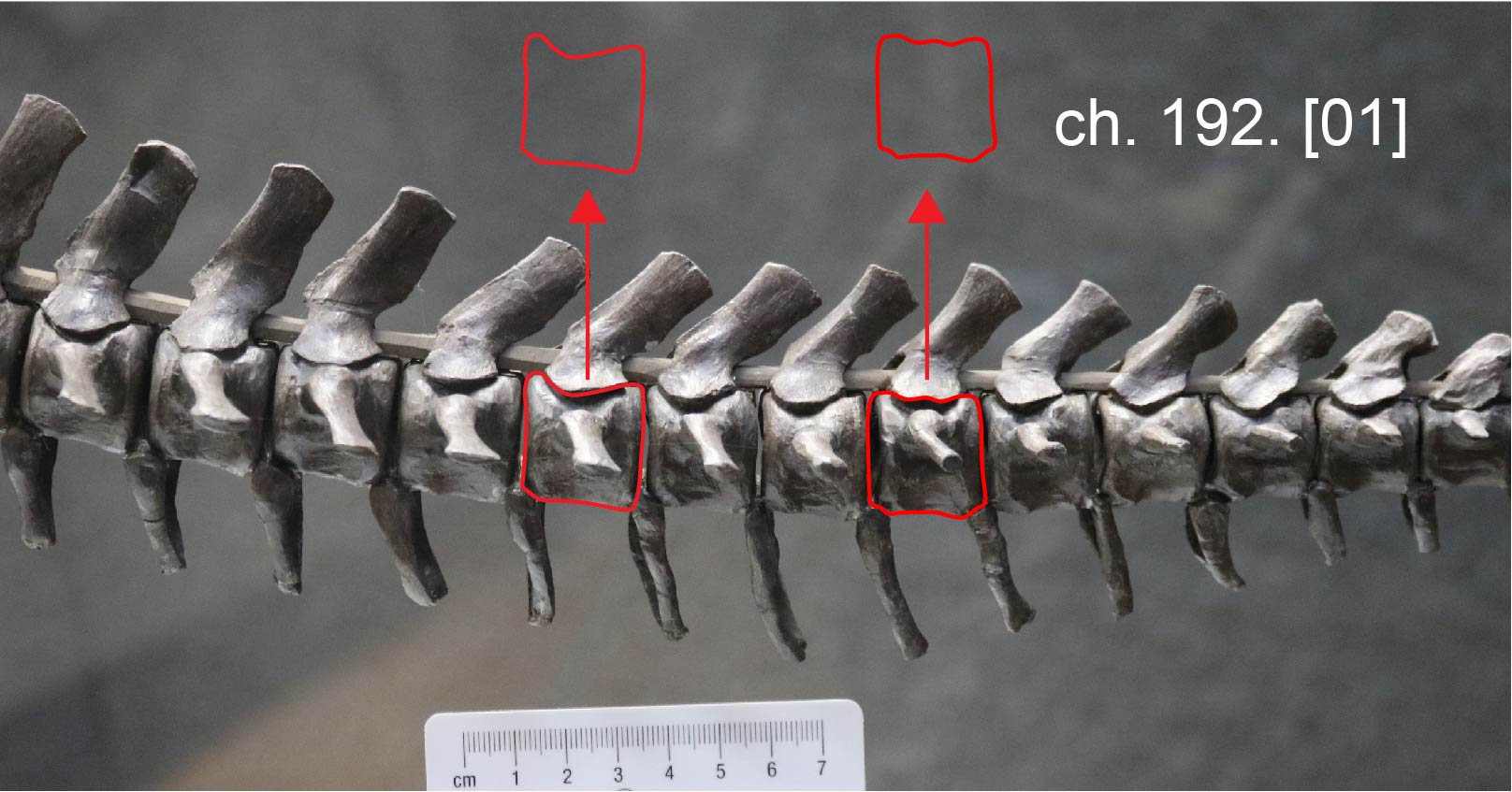


Character 192: state 0 changed to [01]; the chevron facet of the caudal vertebrae is located primarily on both the anterior and posterior faces of some caudal centra, and primarily on the posterior face of other caudal centra.


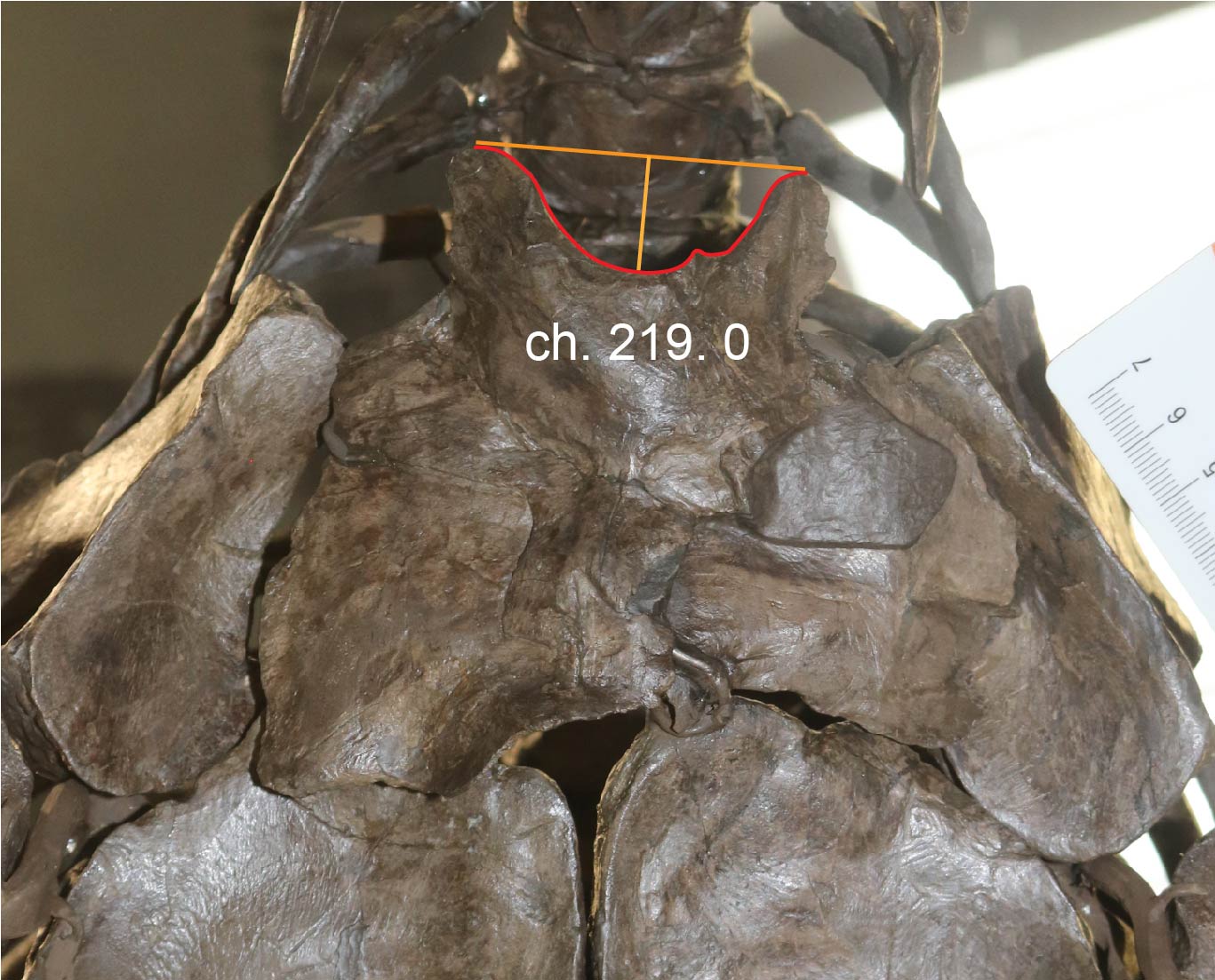


Character 219: state 3 changed to 0; the anterior margin of the clavicle/interclavicle complex is deeply concave with a width at least 1.25 times the anteroposterior depth.


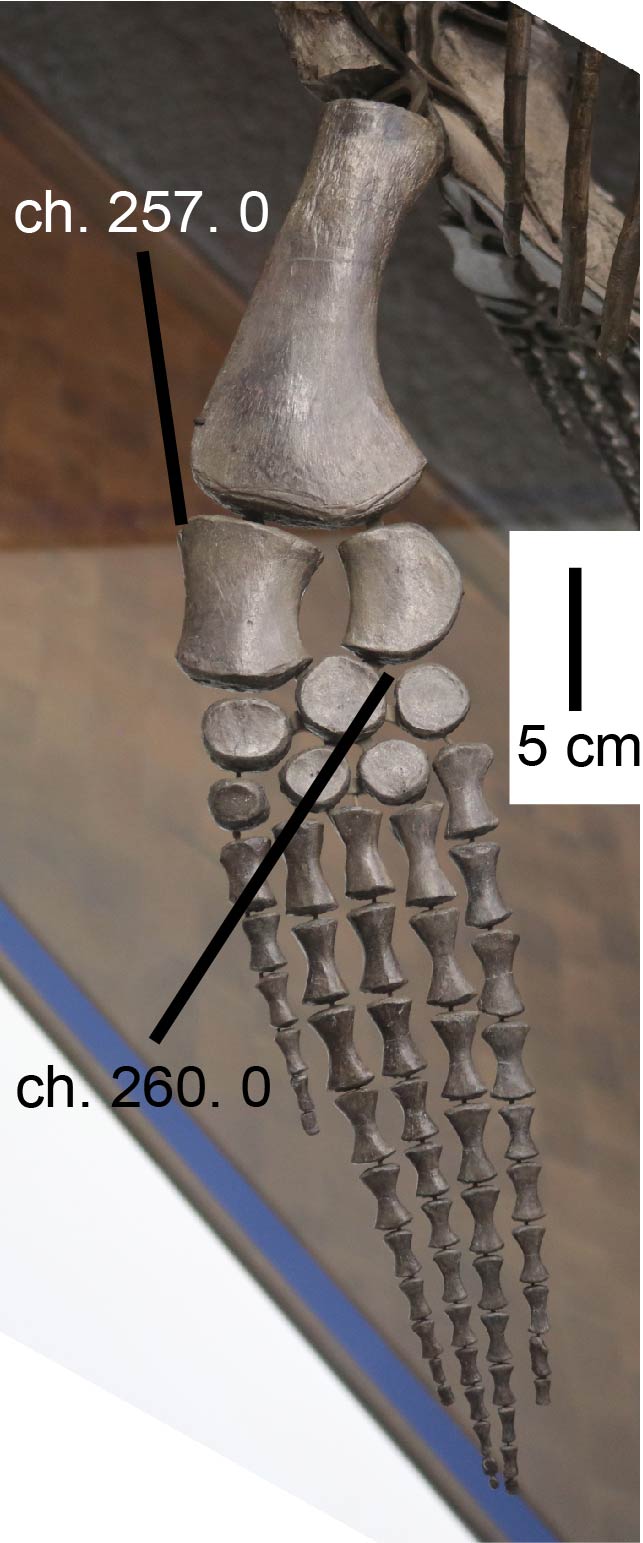


Character 257: state 1 to 0; a prominent anterior flange noes not extend from the anteroproximal face of the radius.

Character 260: state 1 to 0; expansion of the distal end of the ulna relative to the shaft is absent or very weak.
