# Supplementary material for: A new specimen of Plesiopterys wildi reveals the diversification of cryptoclidian precursors and possible endemism within European Early Jurassic plesiosaur assemblages
Source: PeerJ. 2025 Mar 31;13:e18960. doi: 10.7717/peerj.18960 (PMC11967415; doi:10.7717/peerj.18960)

**Consensus tree of 131 taxa (unweighted)**


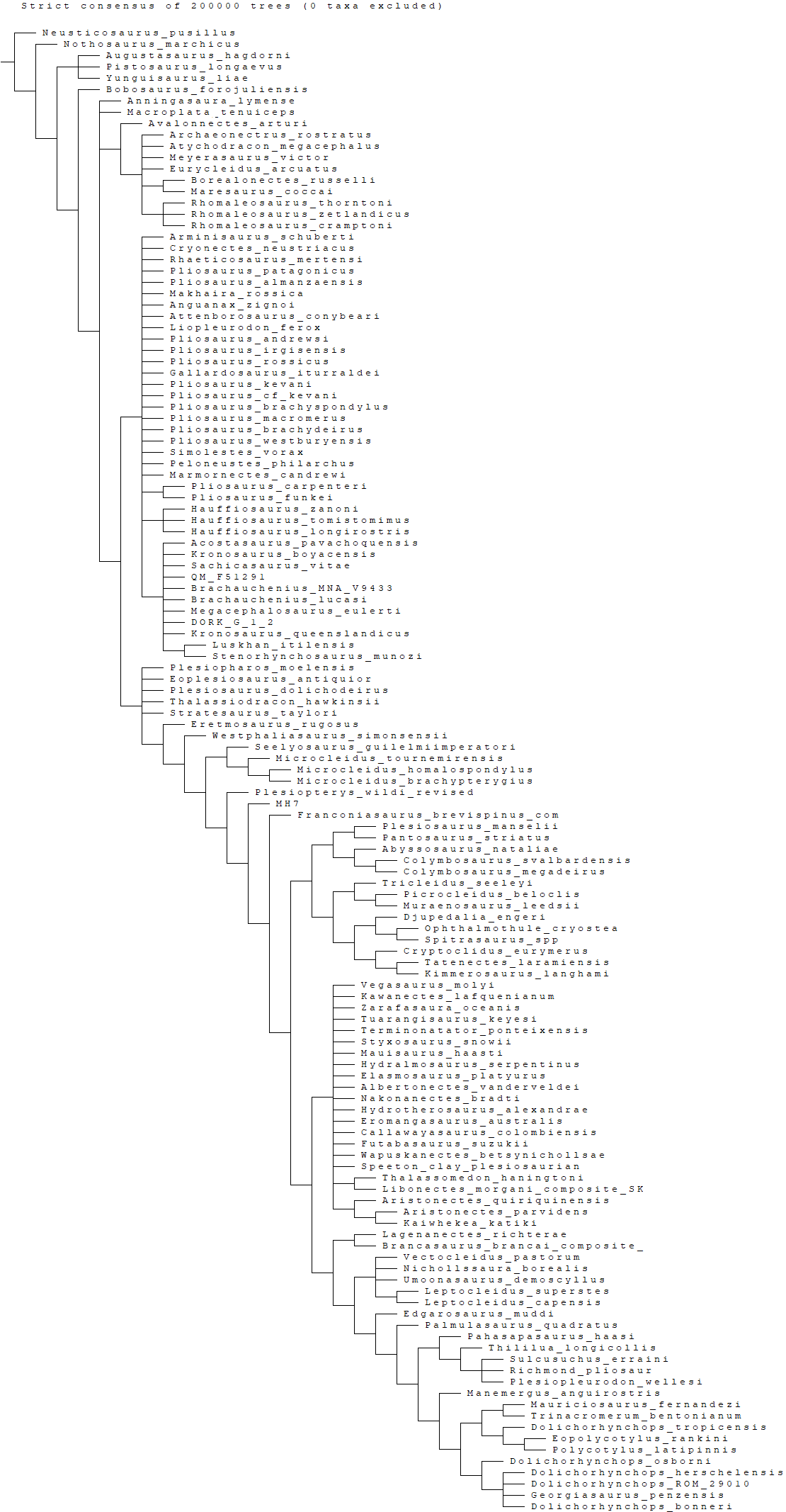


**Consensus tree of 131 taxa (unweighted) with bremer supports**


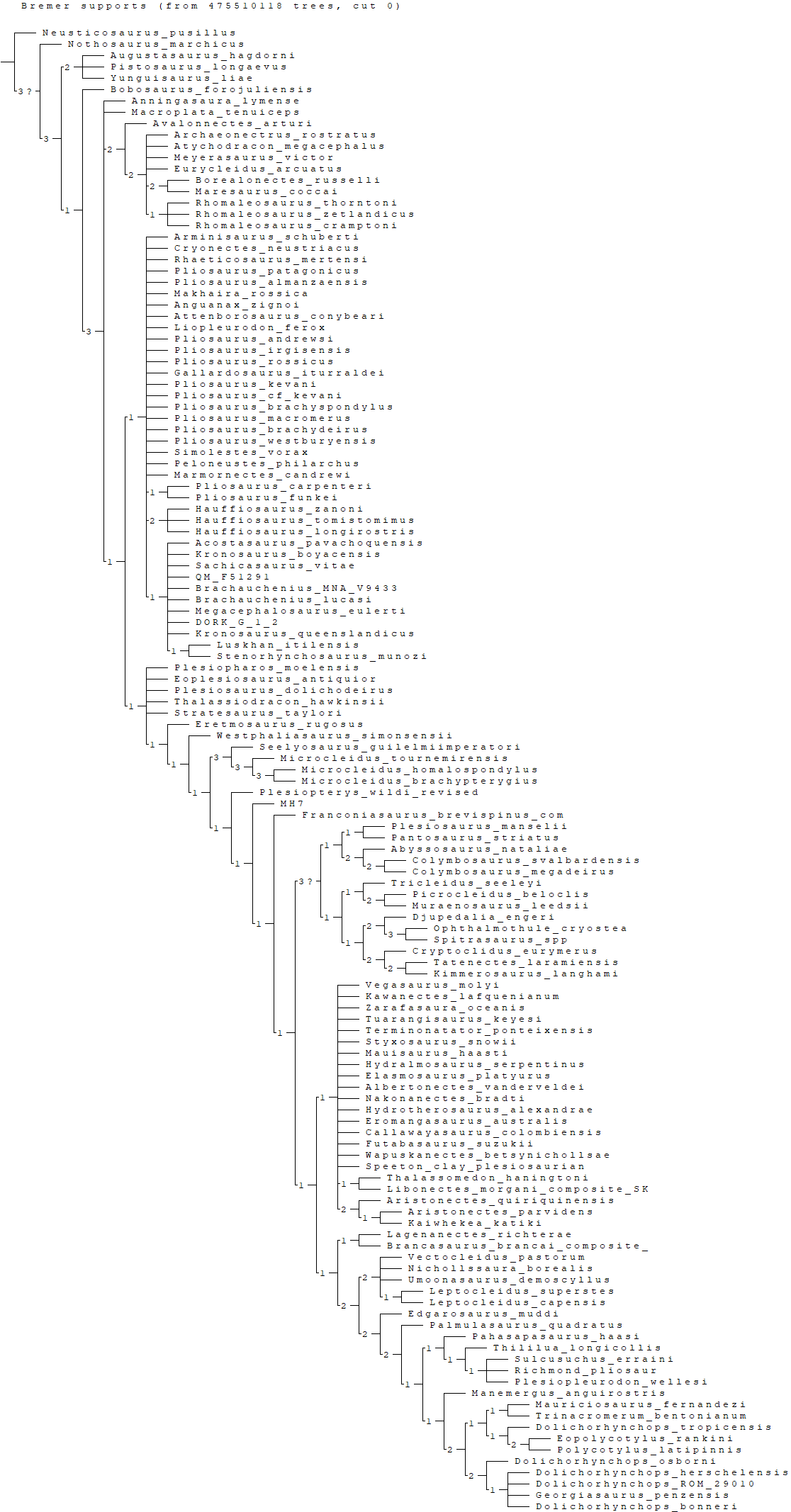


**Consensus tree of 130 taxa (unweighted; SMNS 16812 excluded)**


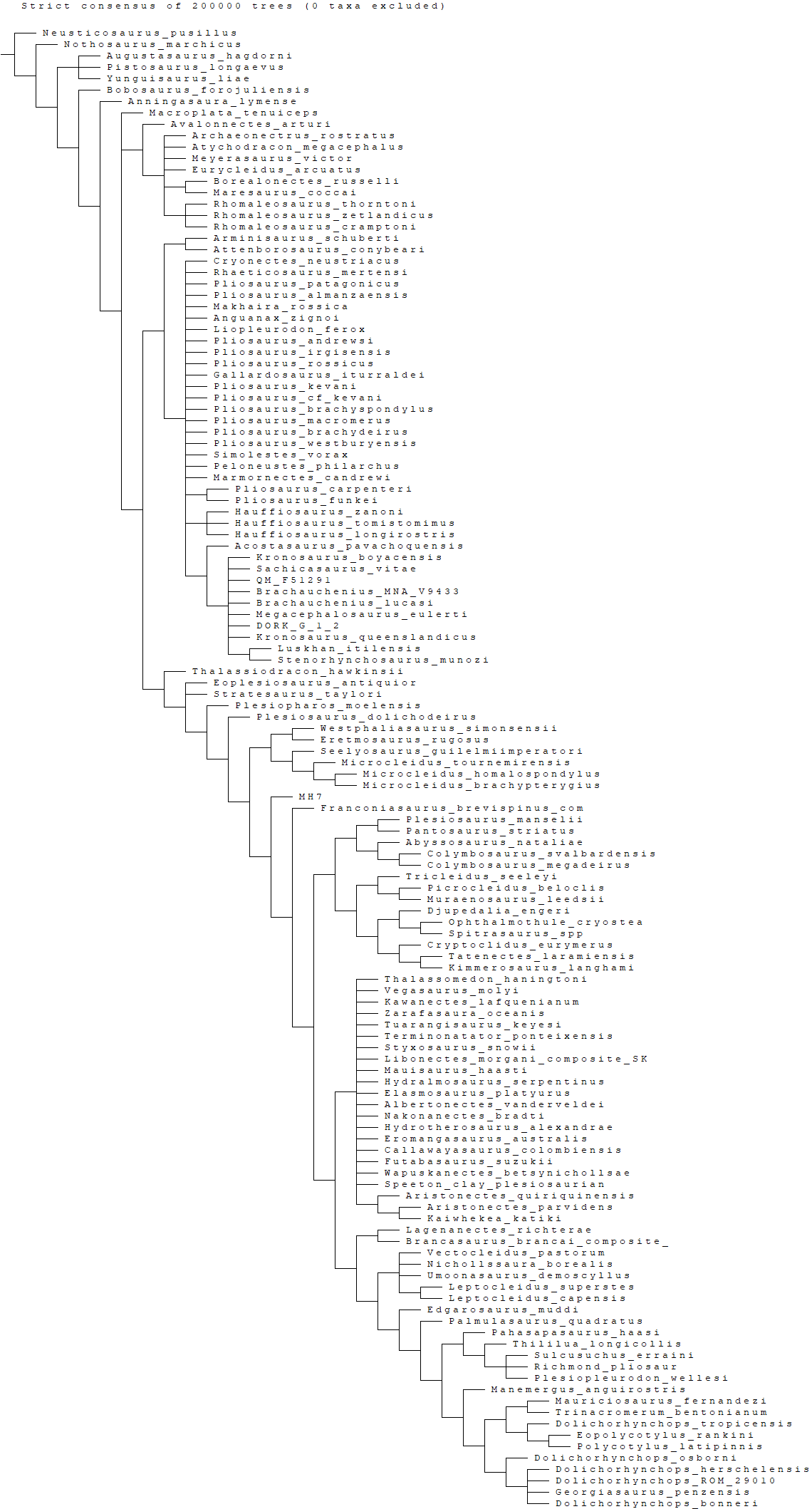


**Consensus tree of 130 taxa (unweighted; SMNS 16812 excluded) with bremer supports**


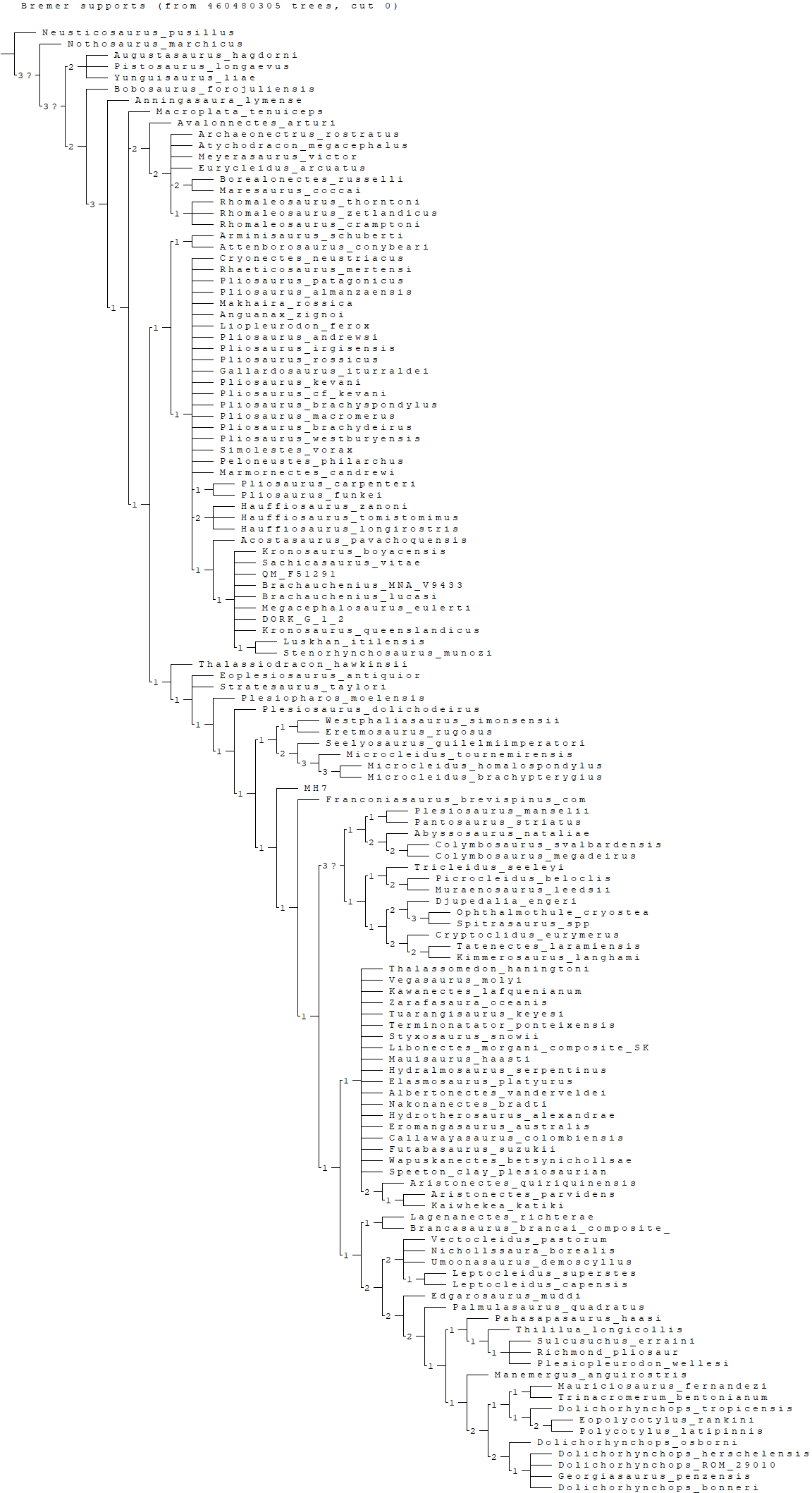


**Consensus tree of 131 taxa (k = 6)**


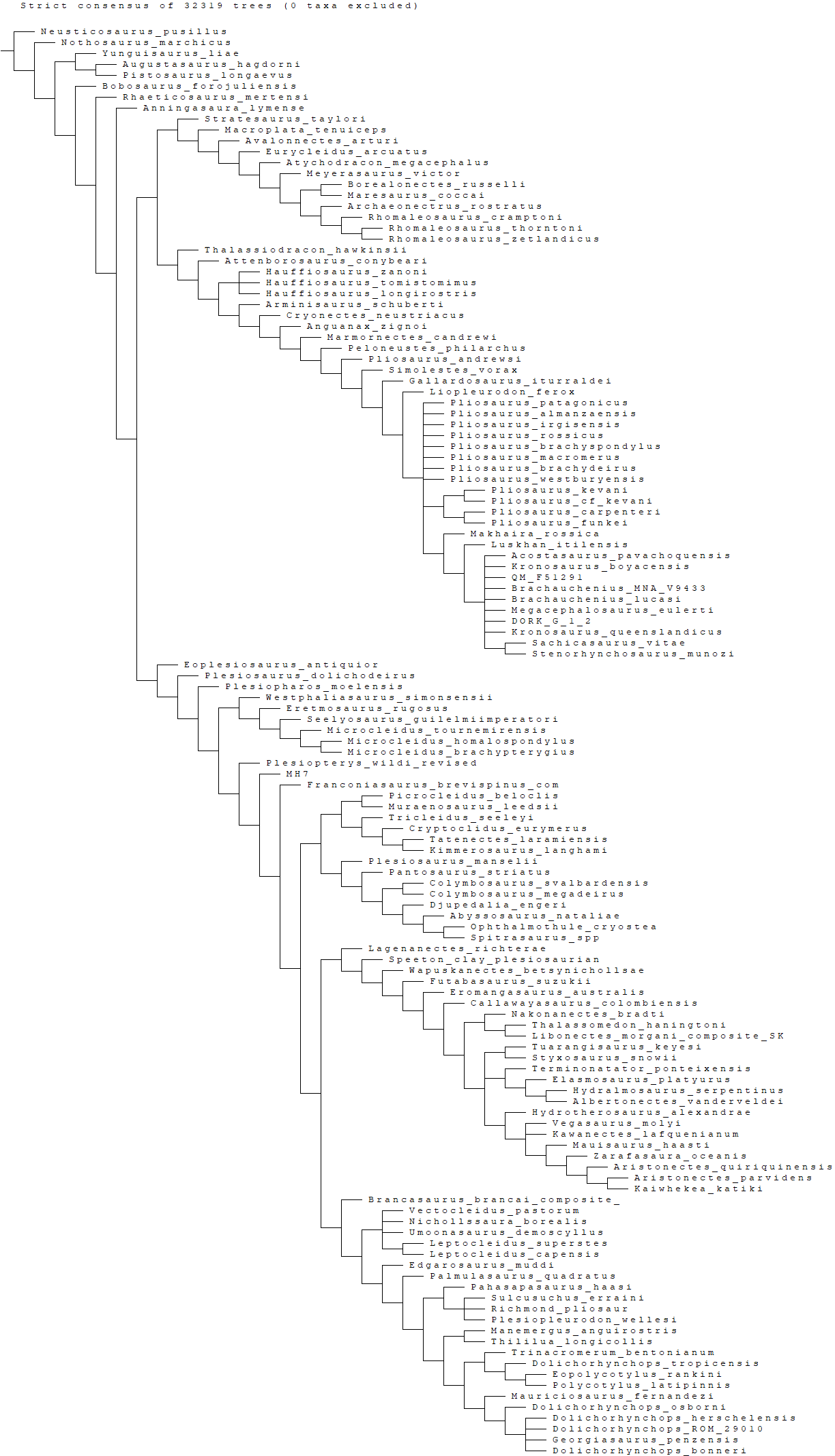


**Consensus tree of 131 taxa (k = 6) with symmetric resampling value supports**


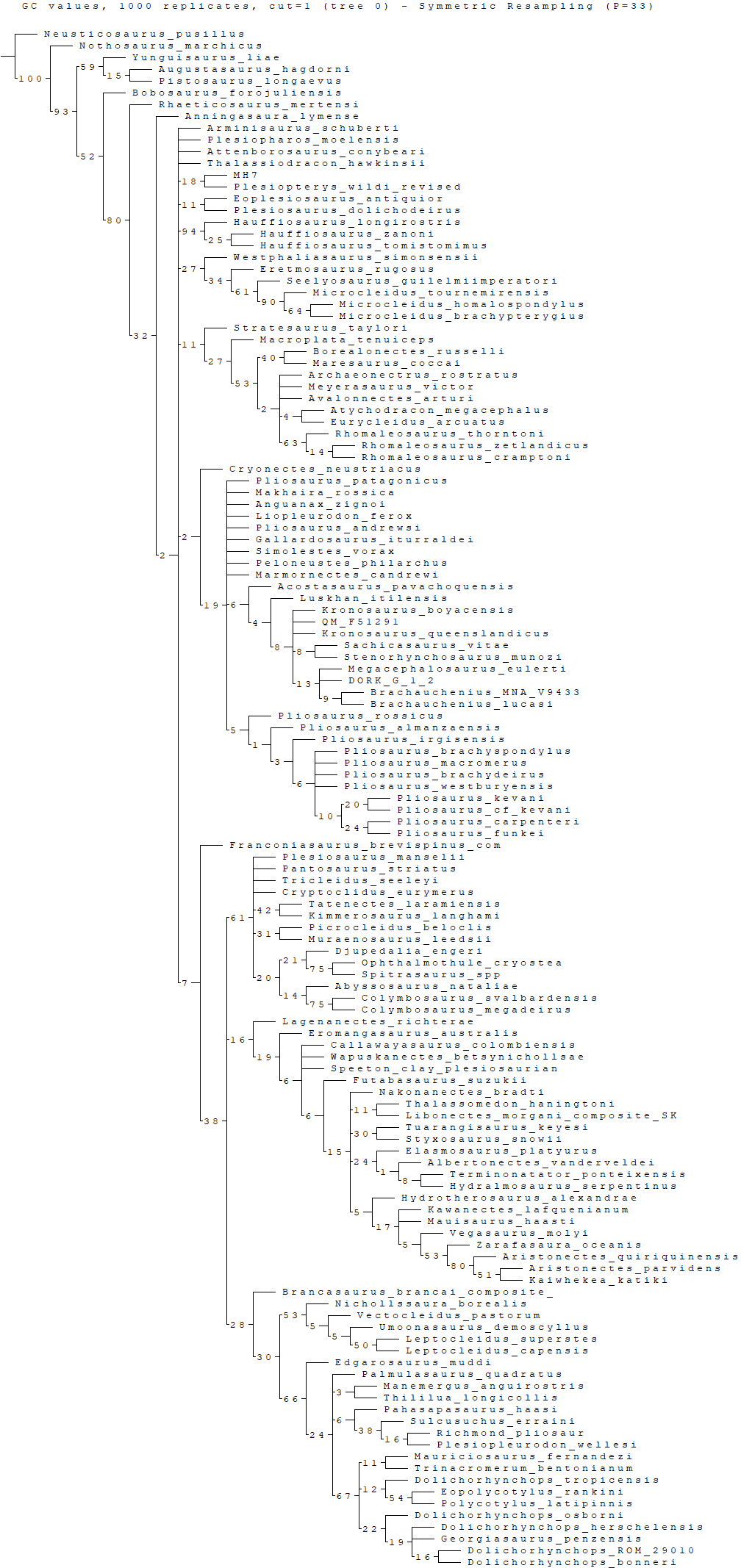


**Consensus tree of 130 taxa (k = 6; SMNS 16812 excluded)**


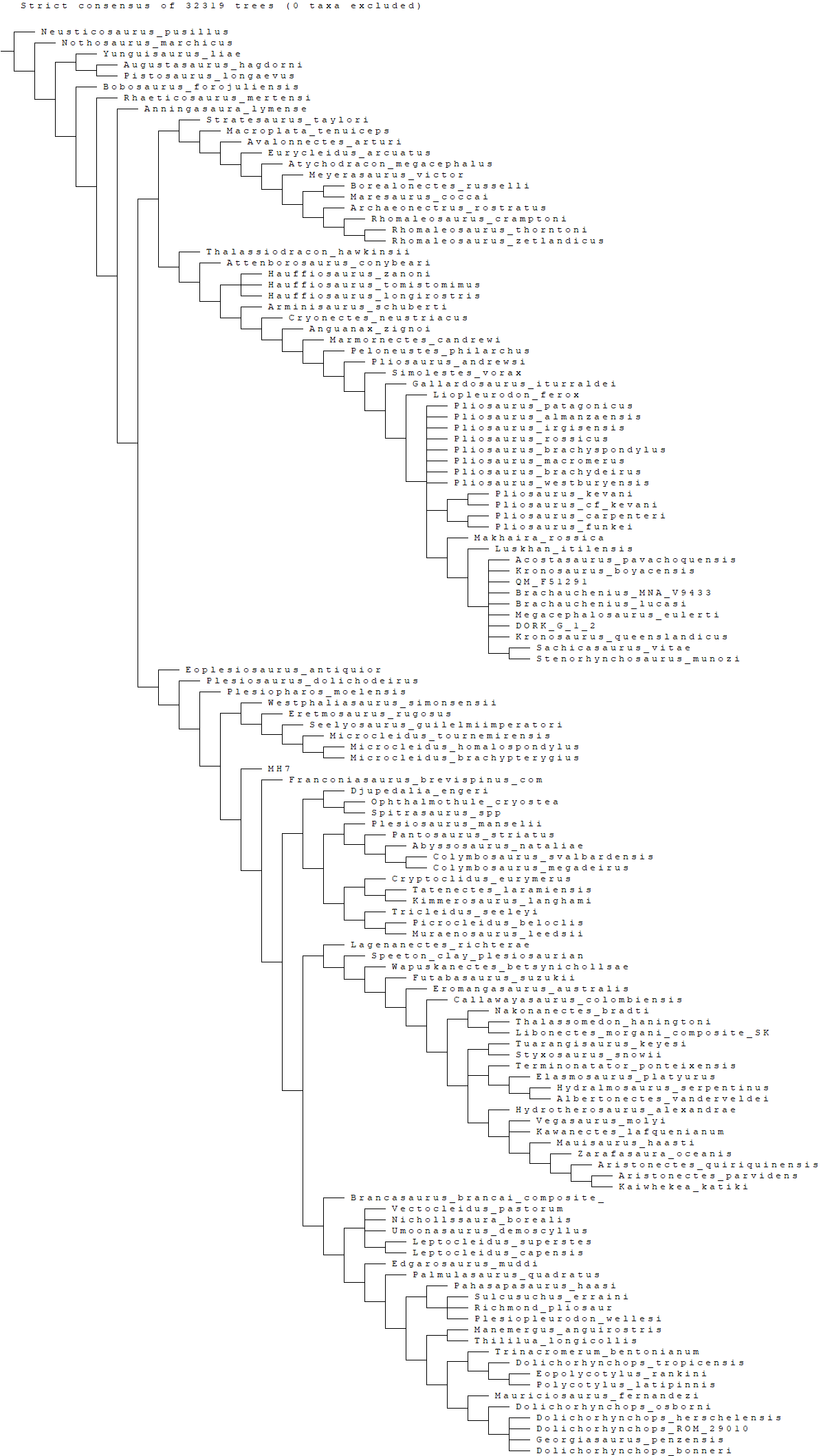


**Consensus tree of 130 taxa (k = 6; SMNS 16812 excluded) with symmetric resampling supports**

**
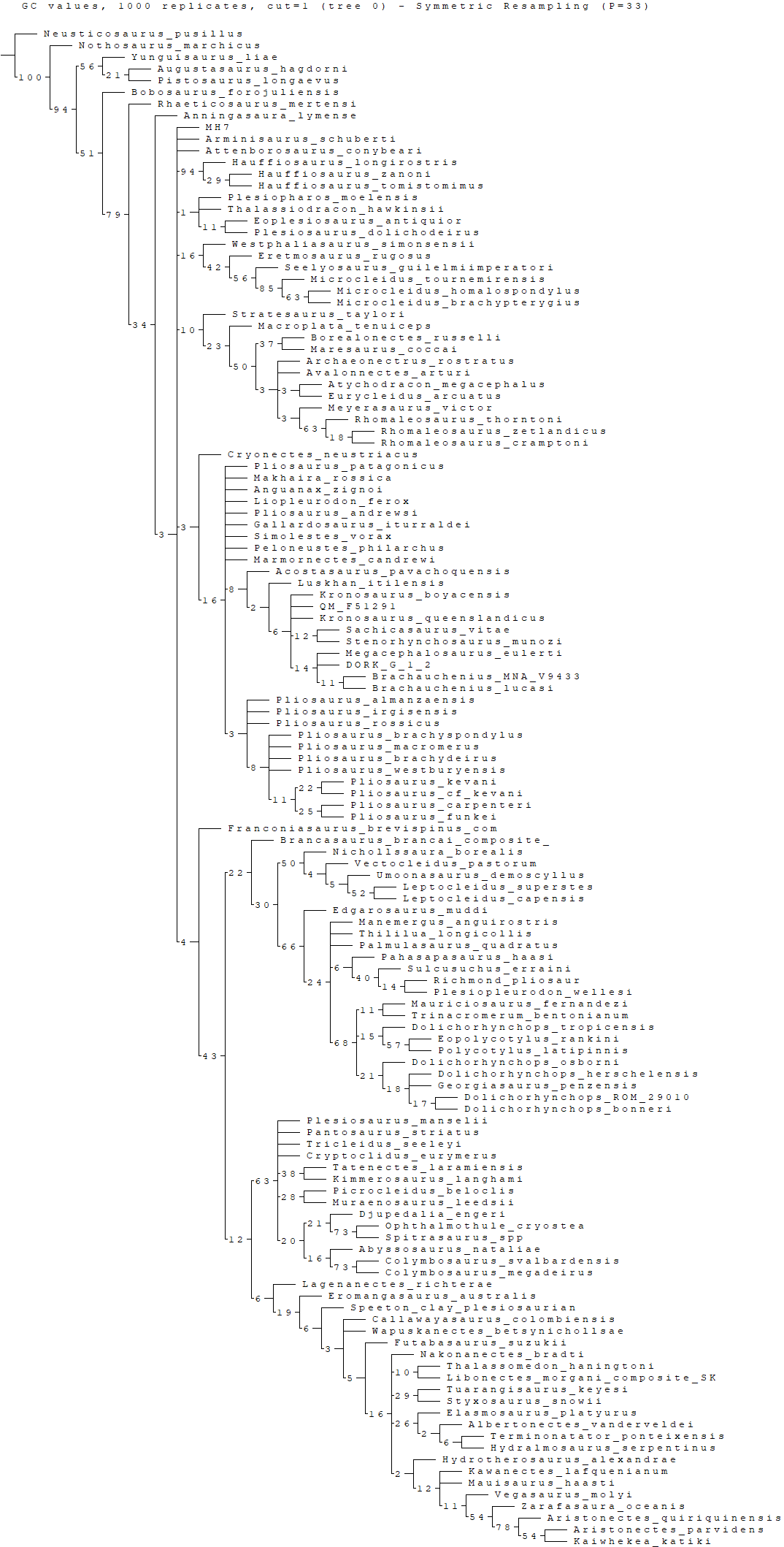
**

**Consensus tree of 131 taxa (k = 9)**


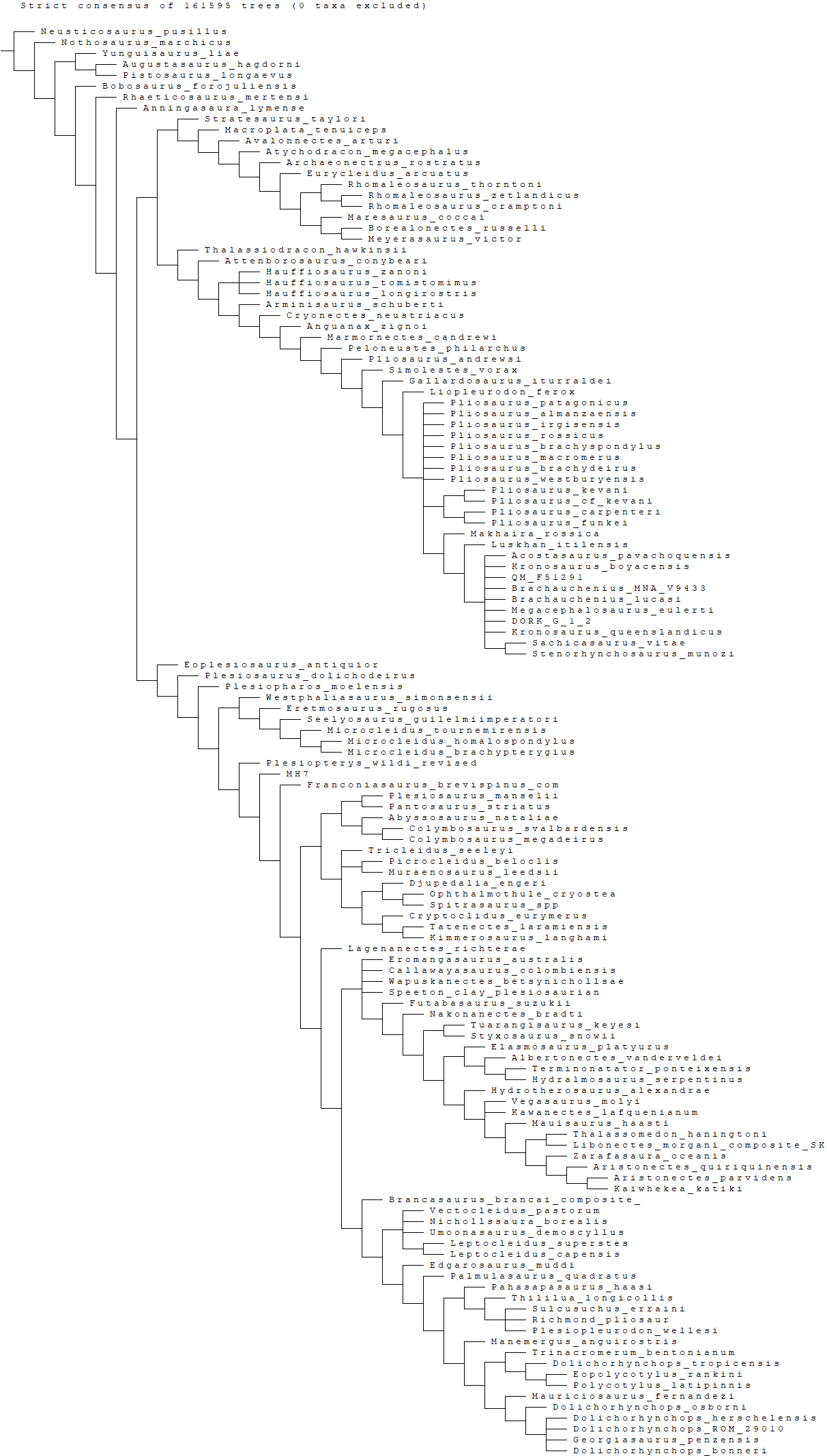


**Consensus tree of 131 taxa (k = 9) with symmetric resampling supports**


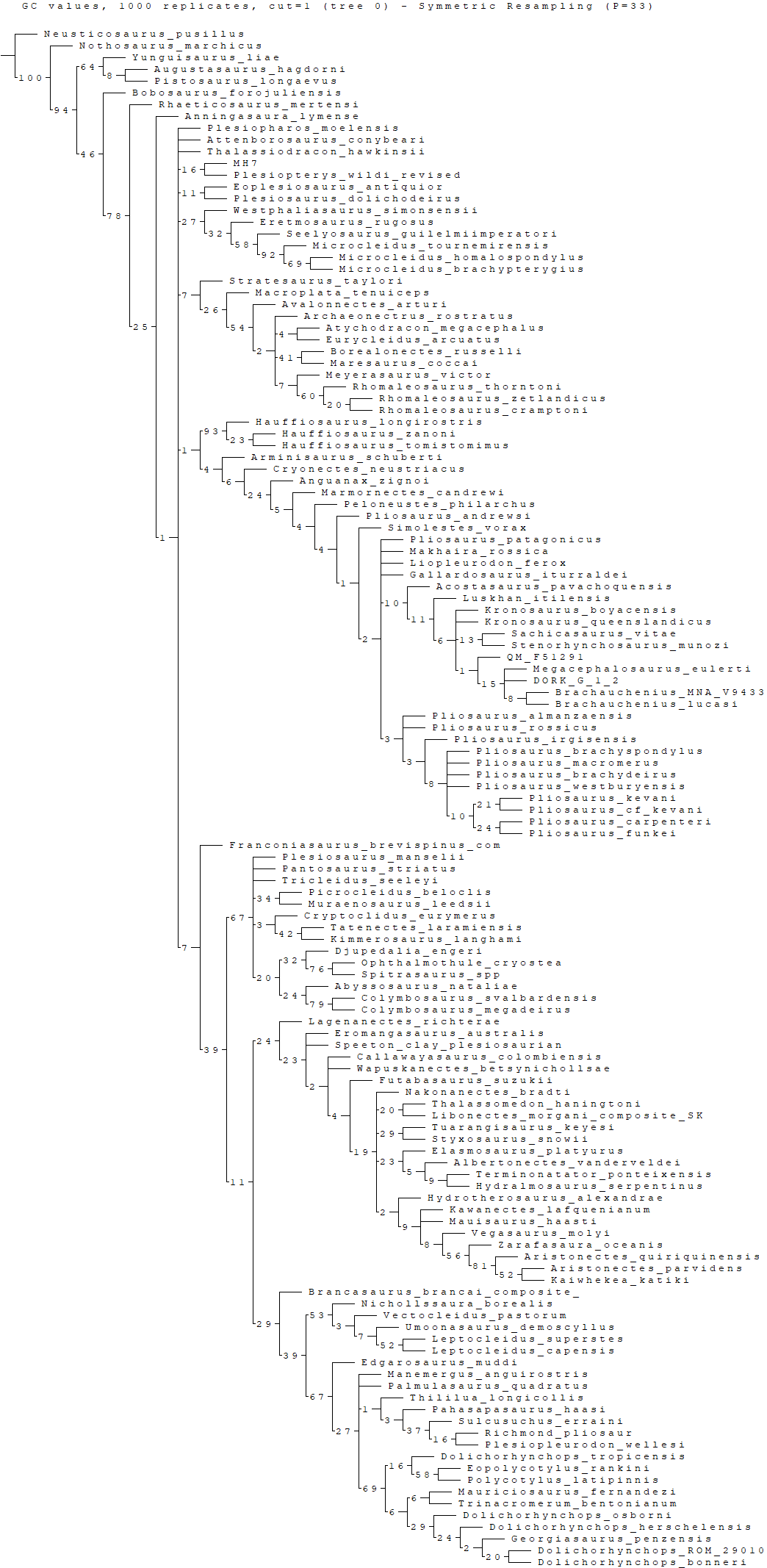


**Consensus tree of 130 taxa (k = 9; SMNS 16812 excluded)**


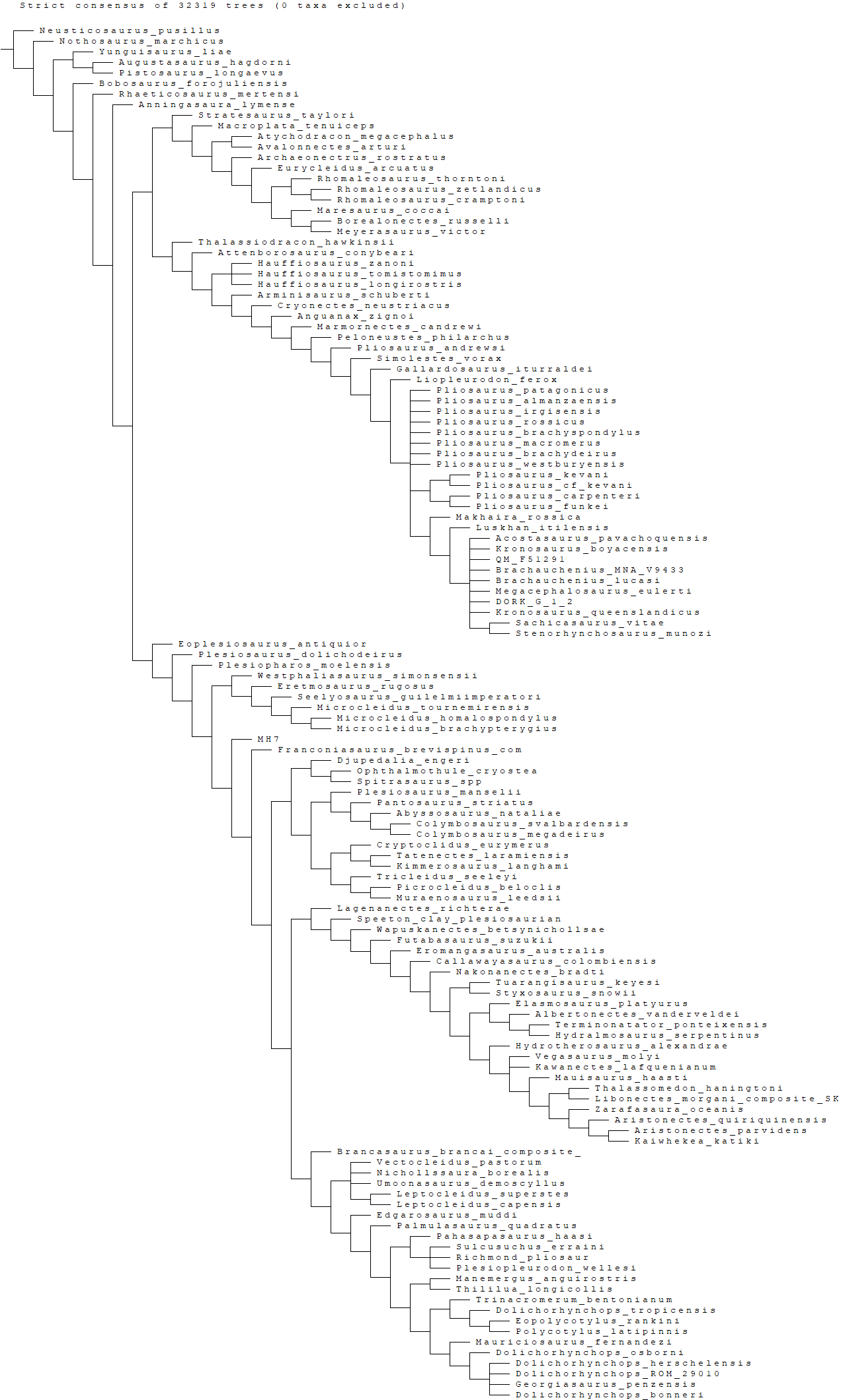


**Consensus tree of 130 taxa (k = 9; SMNS 16812 excluded) with symmetric resampling supports**


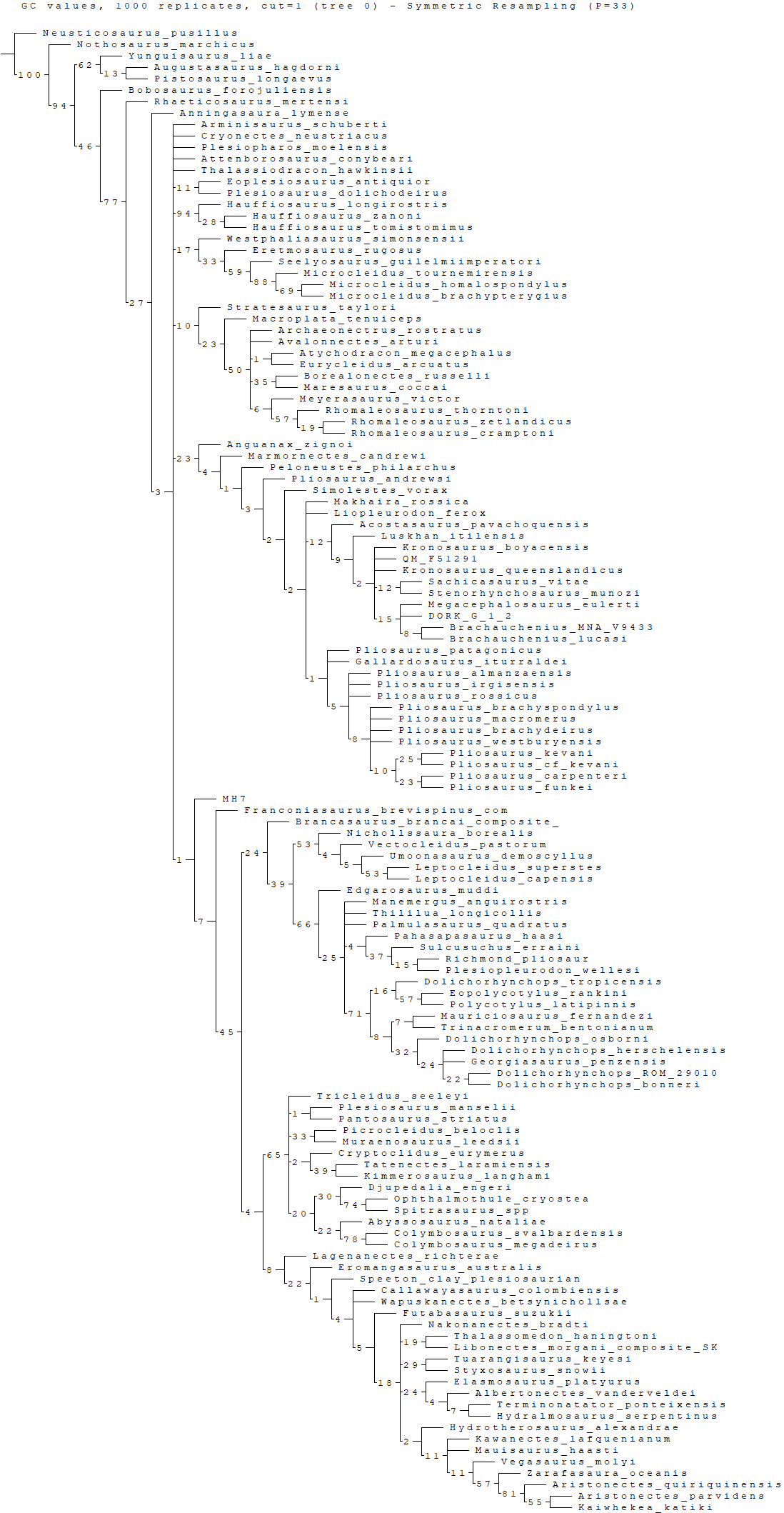

Supplement: Supplemental Information 5 — All of our consensus trees from our six phylogenetic analyses (both weighted and unweighted) with bemer indices and symmetric resampling values reported at the nodes. [file peerj-13-18960-s005.docx]
